# An interaction and feedback mechanism-based group decision-making for emergency medical supplies supplier selection using T-spherical fuzzy information

**DOI:** 10.1038/s41598-023-35909-8

**Published:** 2023-05-30

**Authors:** Shahid Hussain Gurmani, Zhao Zhang, Rana Muhammad Zulqarnain, Sameh Askar

**Affiliations:** 1 School of Mathematical Sciences, Zhejiang Normal University, Jinhua, 321004 Zhejiang China; 2 Department of Mathematics, University of Management and Technology, Sialkot Campus, 51310 Pakistan; 3 Department of Statistics and Operations Research, College of Science, King Saud University, P.O. Box 2455, Riyadh, 11451 Saudi Arabia

**Keywords:** Natural hazards, Mathematics and computing

## Abstract

Selecting a supplier for emergency medical supplies during disasters can be considered a typical multiple attribute group decision-making (MAGDM) problem. MAGDM is an intriguing common problem that is rife with ambiguity and uncertainty. It becomes much more challenging when governments and medical care enterprises adjust their priorities in response to the escalating problems and the effectiveness of the actions taken in different countries. As decision-making problems become increasingly complicated nowadays, a growing number of experts are likely to use T-spherical fuzzy sets (T-SFSs) rather than exact numbers. T-SFS is a novel extension of fuzzy sets that can fully convey ambiguous and complicated information in MAGDM. The objective of this paper is to propose a MAGDM methodology based on interaction and feedback mechanism (IFM) and T-SFS theory. In it, we first introduce T-SF partitioned Bonferroni mean (T-SFPBM) and T-SF weighted partitioned Bonferroni mean (T-SFWPBM) operators to fuse the evaluation information provided by experts. Then, an IFM is designed to achieve a consensus between multiple experts. In the meantime, we also find the weights of experts by using T-SF information. Furthermore, in light of the combination of IFM and T-SFWPBM operator, an MAGDM algorithm is designed. Finally, an example of supplier selection for emergency medical supplies is provided to demonstrate the viability of the suggested approach. The influence of parameters on decision results and comparative analysis with the existing methods confirmed the reliability and accuracy of the suggested approach.

## Introduction

Disasters are the result of great ecological damage between human beings and nature. Collaboration, leadership, and management play a fundamental role in coping with disasters, which may be divided into natural and man-made categories^1,2^. The literature suggests that a disaster might be divided into four stages: mitigation, preparedness, response, and recovery, which stand for reducing or eradicating the likelihood of catastrophe, preparing for it, acting immediately to get people to safety, and returning to normal circumstances. Therefore, emergency management has to be given additional thought in order to limit and manage crisis^3^. It is possible to handle health-related emergencies by taking into account many facets of knowledge, behavior, the medical system, and the system of public health. Additionally, the functionality and setup of the emergency management systems should be considered^4^. Epidemics, earthquakes, tsunamis, tornadoes, wildfires, and other natural disasters are becoming more frequent and severe in developing nations in the twenty-first century, with disastrous effects on people's health, economies, and societies^5–8^. Due to the increase in disasters, humanitarian efforts are also of utmost importance. After these disasters, it is crucial to quickly and precisely reach emergency rescue services and supplies. Researchers in several countries have shifted their focus to finding effective ways to mobilize emergency logistics distribution systems before and after a catastrophe, ensuring proper and timely delivery of supplies to victims^9,10^.

Emergency medical supplies (EMS) are on the frontline between the healthcare systems and people in emergencies and disasters. EMS will be distant from the need if they are not pre-positioned in advance. Zhang et al. explained that emergency medical stocks can play a more important role in reducing causalities instead of allocating emergency medical supplies during an emergency response phase^11^. In^9,12^, scholars have discussed the pre-positioning and pre-deployment of EMS by means of enhancing preparedness for natural disasters. When these situations occur, we need to provide a workable emergency decision-making method using the necessary decision tools to reduce damage and safeguard property and human life^13^.

The suppliers also play an important role in providing the supplies during emergencies. Suppliers are responsible for providing relief supplies to disaster-affected areas, regardless of whether they were procured or donated before or after the disaster. The significance of a disaster can be decreased by choosing an acceptable supplier and establishing contracts on the preparation of medical supplies in advance due to the fact that these actions deliver timely emergency supplies in a specified amount and quality^14,15^. Numerous scholars have conducted research on emergency decision-making, emergency supplier selection, emergency supply chain management, and other related topics. Various factors have to be addressed while selecting any supplier for such issues^16^. There are two main approaches to deal with such type of issues; qualitative analysis and quantitative analysis^17–19^. The selection of suppliers for EMS through a group of experts makes the problem an MAGDM problem. The MAGDM technique, which is the usual and popular method of the third kind, is one of them that takes into account both quantitative and qualitative assessment criteria at the same time in real-life issues. Many factors in an emergency scenario are challenging to quantify and assess. The MAGDM approach may thus be more persuasive than other ways to some extent and the very common method for supplier selection problems which has been used by many scholars in various real-life problems^12,16,20–22^. A rising number of academics have used the MAGDM technique to tackle emergencies in recent years^23^. However, due to issues like high expense, prolonged delay, and poor emergency decision-making efficiency, it is challenging to ensure the speed, application, and quality of relevant materials, which increases the complexity of this method's process^24,25^. However, scholars have rarely thought about the intricate link between these aspects. The existing MAGDM supplier selection techniques are ineffective in emergency issues. Additionally, while making judgments, DMs may have diverse risk thresholds, which causes significant difficulties for scholars when deciding which emergency medical supplier to choose^24^.

Multiple attribute group decision-making (MAGDM) is a decision-making process that involves a group of decision-makers who evaluate multiple options based on a set of criteria or attributes. The objective of MAGDM is to select the best option among several alternatives, taking into account the preferences of several decision-makers. In MAGDM, decision-makers provide their opinions or judgments on the importance of various attributes and the performance of different alternatives. These judgments can be in the form of numerical ratings, verbal descriptions, or rankings. The judgments are then aggregated using a decision-making method to drive a consensus decision. In many domains of engineering, management, and other decision-making, when just a few options are available and the best option must be chosen, MAGDM techniques are essential. Due to competing trade-offs across criteria, decision-making in the public health system is challenging, and single-attribute decision-making techniques fail to accurately model issues when the decision-making environment becomes more complicated. In terms of the ways that people naturally make decisions, MAGDM techniques are more intuitive. Modeling human behavior with MAGDM is rapidly evolving in various fields^26–30^.

However, due to the ambiguity of things and the uncertainty of situations, the key issue of experts in MAGDM is dealing with how to efficiently acquire assessment information. Thus, to deal with such scenarios, Zadeh proposed a fuzzy set in 1965 that described the ambiguity of objects with membership degree (MD) having a value between zero and one^31^. The development of several fields has been boosted by the appearance of FSs to determine a clear solution to more complex decision-making issues^32^. After an improved form of FSs described by Zadeh^33^ in 1975, Atanassov^34^ expanded the fuzzy sets into intuitionistic fuzzy sets (IFSs), represented with membership and non-membership degrees and satisfy that the total of MD and non-membership degree (NMD) does not exceed one. Further extension of FSs and IFSs is called Pythagorean fuzzy sets (P_y_FSs) proposed by Yager^35^, characterized by MD and NMD providing the condition that the square sum of MD and NMD must lie between zero and one. A broader class of IFSs and P_y_FSs is later presented by Yager^36^ in the form of q-rung orthopair fuzzy sets (q-ROFSs). The MD and NMD of an element in a fuzzy set are used to describe q-ROFSs, and the sum of their qth powers must be at most 1. Yager stated that when ‘q’ increases, the number of admissible orthopairs expands, giving experts more freedom than previous fuzzy set extensions to express their opinions on membership degree. The development of these extended fuzzy sets mentioned above has drawn the attention of several academics for the past few decades and has found usage in a variety of disciplines^37–39^.

Nevertheless, in many daily life situations, the utilization of FSs, IFSs, P_y_FSs, and q-ROFSs may lead to the loss of evaluation information. For instance, when voting, people's attitudes may include hesitation and rejection in addition to yes and no. Cuong^40^ introduced picture fuzzy set (PFS) to address this issue. These PFSs captured the vagueness of objects in a more thorough manner from four aspects: MD, abstinence degree (AD), and NMD, and satisfied the condition that the sum of these must not be greater than 1. PFSs are undoubtedly better at describing ambiguity than FSs, IFSs, P_y_FSs, and q-ROFSs. Recent years have seen the development of various PFS investigations, such as^41–44^. Even though PFSs are an effective mathematical tool for decision-making, they cannot resolve this scenario where the sum of MD, AD, and NMD exceeds one. For example, if any expert provides his/her assessment in the form of picture fuzzy number as, $MD = 0.7$, $AD = 0.3$ and $NMD = 0.4$, according to the condition of PFSs, it can be observed that $0.7 + 0.3 + 0.4 = 1.4 > 1$. In this situation, the PFSs failed. To tackle this type of issue, Mahmood et al.^45^ came up with the inception of spherical fuzzy sets (SFSs), which is capable of dealing with the above-mentioned shortcomings and satisfy the condition that the square sum of MD, AD, and NMD must lie between zero and one. According to the above example we can see that $\left( {0.7} \right)^{2} + \left( {0.3} \right)^{2} + \left( {0.4} \right)^{2} = 0.74 < 1$; the condition for SFSs holds. However, sometime the SFSs are also unable to solve the problems when the sum of MD, AD, and NMD exceeds one, such as if $MD = 0.7$, $AD = 0.5$, $NMD = 0.6$, $\left( {0.7} \right)^{2} + \left( {0.5} \right)^{2} + \left( {0.6} \right)^{2} = 1.1 > 1$. In this situation, further expansion of the existing structure of SFS was provided in the same study which is called T-spherical fuzzy set (t-SFS). The representation of a parameter ‘q’ into the constraints such that qth sum of MD, AD, and NMD must not exceed one; then then by taking the value of $q \ge 3$, the above example $\left( {0.7} \right)^{3} + \left( {0.5} \right)^{3} + \left( {0.6} \right)^{3} = 0.684 < 1$ holds. If the value of parameter is one and two, then t-SFSs are reduced to PFSs and SFSs, respectively. T-spherical fuzzy sets with zero abstinence degrees are mathematically equivalent to q-ROFSs. On the other hand, t-SFSs with null abstinence degrees are considered as generalized form of IFSs and P_y_FSs in cases where parameter is one and two, respectively. SFSs and T-SFSs are now the focus of decision system research. For example, in^46^, the authors designed and extended the MULTMOORA method using SFSs. Garg^47^ proposed the power aggregation operators under T-SF information. Yang et al.^48^ developed a novel information error-driven T-spherical fuzzy cloud method to assess small and medium-sized businesses' innovative digital transformation strategies. Debnath et al.^49^ defined power-partitioned neutral aggregation operators for T-SFSs. Furthermore, T-SFSs have been used in MAGDM methods recently. Ullah et al. designed a correlation coefficient-based MAGDM technique using T-SFSs and used it to solve the clustering problem^50^. In^51^, T-SF Power Muirhead mean operator-based MAGDM is established to tackle the decision problem of daily life. Researchers who are interested are directed to some decent literature on MAGDM using T-SFSs^52–57^.

This analysis of the literature revealed that it might be challenging for the MAGDM method-based research to effectively capture the features of emergency decision-making. However, there is still a dearth of MAGDM exploration on the assessment of EMS supplier selection, and the relevant literature still suffers from several problems, broadly categorized into three types: (1) The prior study on emergency decision-making did not take into consideration the interaction of experts with each other which is obvious these days. Realistic circumstances frequently include complex causal relationships between several factors. Simultaneously, the expert’s preferences, educational background, and consensus level are always different. Therefore, it could be challenging for current MAGDM approaches to effectively capture the incomplete reasoning of experts and the peculiarities of the criteria used to select the suppliers for emergency medical supplies^18^. (2) Few academics have thoroughly thought about how to balance evaluation criteria in emergency decision-making, such as subjective and objective elements, weights of experts and criteria, etc. The accuracy of the results may be impacted by the single-weighting method's ability to strengthen or reduce the influence of various elements^58,59^. (3) Numerous MAGDM research that has already been conducted on decision-making use fuzzy sets as their foundation, sometimes using extended fuzzy sets that use the membership degree and non-membership degree to describe the assessment of experts. For example, when the experts don’t want to give any response about any object, then neutrality is involved. The literature about the involvement of neutrality in emergency decision-making is not available. When choosing a supplier for emergency medical supplies, these fuzzy sets may have numerous drawbacks that are very obvious, which can lead the experts to an erroneous assessment procedure.

In order to fill this research gap, our analysis suggests an interaction and feedback mechanism-based MAGDM methodology using T-SFSs to get the group consistency between multiple experts. The proposed analysis framework is novel as it is based on T-SF information using interaction and feedback mechanism to get the group consistency between experts for EMS supplier selection. In the same time, the weights of experts are obtained by using the consistency degree and hamming distance measure. This study makes five major contributions which are as follows: (1) Considering the partitioned structure among criteria, we extend the partitioned Bonferroni mean operator to accommodate the T-spherical fuzzy environment, and two aggregation operators are developed to integrate the evaluation information of experts, namely, T-spherical fuzzy partitioned Bonferroni mean operator and T-spherical fuzzy weighted partitioned Bonferroni mean operator. (2) We improve the emergency decision-making process using a unique quantitative weighting method to find the weights of experts. (3) In order to overcome the difficulty of selecting emergency medical supplies suppliers, we propose a decision-making framework to get the final alternative ranking by considering interaction and feedback mechanism to disclose the conflicts between experts while providing their assessments. This framework reconciles differing expert opinions in light of the limitations of existing studies and the complex nature of decision problems. (4) To demonstrate the viability of the designed algorithm, the advised technique is utilized to process a supplier selection example for emergency medical supplies. (5) Finally, the sensitivity analysis confirms that the suggested technique is robust, and the comparison with the current approaches further demonstrates the suggested method's efficacy and superiority.

The study is structured as follows: "Preliminaries" section elaborates on the basic knowledge of T-SFSs, including its definition, basic operations, score and accuracy functions, Bonferroni mean operator, and partitioned Bonferroni mean operator. In "T-spherical fuzzy partitioned Bonferroni mean operator" section, two aggregation operators are introduced, and some of their prominent properties are discussed. "Group decision-making framework based on IFM and aggregation operators for T-SFN" section presents a group decision-making framework based on IFM and aggregation operators for T-SFN. In “Numerical example and comparative analysis” section, the methodology proposed in the previous section is applied to an actual instance to obtain ranking results, and the results are examined from two aspects. The section also includes a comparative analysis of the proposed methodology with existing methods. Finally, "Conclusion" section provides a brief conclusion of the study.

## Preliminaries

In this section, some basic concepts related to T-SFSs are recalled to better understand the research ahead.

### **Definition 2.1**

^31^ Let $U$ be a fixed set. Fuzzy set (FS) ‘$F$’ on $U$ can be represented as

$$F = \left\{ {\left( {\upsigma ,\mu_{F} \left(\upsigma \right)} \right)\left| {\upsigma \in U} \right.} \right\}$$

where $\mu_{F} \left(\upsigma \right):U \to \left[ {0,1} \right]$ is called membership of the element $\upsigma \in U$ to the set $F$.

### **Definition 2.2**

^35^ Let $U$ be a fixed set. Pythagorean fuzzy set (P_y_FS) ‘$P$’ on $U$ can be represented as

$$P = \left\{ {\left( {\upsigma ,\mu_{P} \left(\upsigma \right),v_{P} \left(\upsigma \right)} \right)\left| {\upsigma \in U} \right.} \right\}$$

where $\mu_{P} \left(\upsigma \right):U \to \left[ {0,1} \right]$ and $v_{P} \left(\upsigma \right):U \to \left[ {0,1} \right]$ are called MD and NMD of the element $\upsigma \in U$ to the set $P$. For every $\upsigma \in U$, it satisfies the following condition: $0 \le \mu_{P}^{2} \left(\upsigma \right) + v_{P}^{2} \left(\upsigma \right) \le 1$. Additionally, $\pi_{P} = \sqrt {1 - \left( {\mu_{P}^{2} \left(\upsigma \right) + v_{P}^{2} \left(\upsigma \right)} \right)}$ is known as the degree of indeterminacy $\upsigma \in U$. For simplicity, $\left( {\mu_{P} \left(\upsigma \right),v_{P} \left(\upsigma \right)} \right)$ is called P_y_F number (P_y_FN) and is denoted as $p = \left( {\mu ,v} \right)$.

### **Definition 2.3**

^36^ Let $U$ be a universal set. q-rung orthopair fuzzy set (q-ROFS) ‘$Q$’ on $U$ can be represented as

$$Q = \left\{ {\left( {\upsigma ,\mu_{Q} \left(\upsigma \right),v_{Q} \left(\upsigma \right)} \right)\left| {\upsigma \in U} \right.} \right\}$$

where $\mu_{Q} \left(\upsigma \right):U \to \left[ {0,1} \right]$ and $v_{Q} \left(\upsigma \right):U \to \left[ {0,1} \right]$ are called MD and NMD of the element $\upsigma \in U$ to the set $Q$. For every $\upsigma \in U$, it satisfies the following condition: $0 \le \mu_{Q}^{q} \left(\upsigma \right) + v_{Q}^{q} \left(\upsigma \right) \le 1$ where $\left( {q \ge 1} \right)$. Additionally, $\pi_{Q} = \sqrt[q]{{1 - \left( {\mu_{Q}^{q} \left(\upsigma \right) + v_{Q}^{q} \left(\upsigma \right)} \right)}}$ is known as the degree of indeterminacy $\upsigma \in U.$ For simplicity, $\left( {\mu_{Q} \left(\upsigma \right),v_{Q} \left(\upsigma \right)} \right)$ is called q-ROF number (q-ROFN) and is denoted as $Q = \left( {\mu ,v} \right).$

### **Definition 2.4**

^45^ Let $U$ be a fixed set. Spherical fuzzy set (SFS) ‘$S$’ on $U$ can be represented as

$$S = \left\{ {\left( {\upsigma ,\mu_{S} \left(\upsigma \right),\eta_{S} \left(\upsigma \right),v_{S} \left(\upsigma \right)} \right)\left| {\upsigma \in U} \right.} \right\}$$

where $\mu_{S} \left(\upsigma \right):U \to \left[ {0,1} \right]$, $\eta_{S} \left(\upsigma \right):U \to \left[ {0,1} \right]$ and $v_{S} \left(\upsigma \right):U \to \left[ {0,1} \right]$ are called MD, AD, and NMD of the element $\upsigma \in U$ to the set $S$. For every $\upsigma \in U$, it satisfies the following condition: $0 \le \mu_{S}^{2} \left(\upsigma \right) + \eta_{S}^{2} \left(\upsigma \right) + v_{S}^{2} \left(\upsigma \right) \le 1$. Additionally, $\pi_{S} = \sqrt {1 - \left( {\mu_{S}^{2} \left(\upsigma \right) + \eta_{S}^{2} \left(\upsigma \right) + v_{S}^{2} \left(\upsigma \right)} \right)}$ is known as the degree of indeterminacy $\upsigma \in U$. For simplicity, $\left( {\mu_{S} \left(\upsigma \right),\eta_{S} \left(\upsigma \right),v_{S} \left(\upsigma \right)} \right)$ is called SF number (SFN) and is denoted as $s = \left( {\mu ,\eta ,v} \right)$.

### **Definition 2.5**

^45^ Let $U$ be a fixed set. T-SFS $A$ on $U$ can be represented as

$$A = \left\{ {\left( {\upsigma ,\mu_{A} \left(\upsigma \right),\eta_{A} \left(\upsigma \right),v_{A} \left(\upsigma \right)} \right)\left| {\upsigma \in U} \right.} \right\}$$

where $\mu_{A} \left(\upsigma \right):U \to \left[ {0,1} \right]$, $\eta_{A} \left(\upsigma \right):U \to \left[ {0,1} \right]$ and $v_{A} \left(\upsigma \right):U \to \left[ {0,1} \right]$ are called MD, AD, and NMD of the element $\upsigma \in U$ to the set $A$. For every $\upsigma \in U$, it satisfies the following condition: $0 \le \mu_{A}^{q} \left(\upsigma \right) + \eta_{A}^{q} \left(\upsigma \right) + v_{A}^{q} \left(\upsigma \right) \le 1$ where $\left( {q \ge 1} \right)$. Additionally, $\pi_{A} = \sqrt[q]{{1 - \left( {\mu_{A}^{q} \left(\upsigma \right) + \eta_{A}^{q} \left(\upsigma \right) + v_{A}^{q} \left(\upsigma \right)} \right)}}$ is known as the degree of indeterminacy $\upsigma \in U$. For simplicity, $\left( {\mu_{A} \left(\upsigma \right),\eta_{A} \left(\upsigma \right),v_{A} \left(\upsigma \right)} \right)$ is called T-SF number (T-SFN) and is denoted as $a = \left( {\mu ,\eta ,v} \right)$.

### **Definition 2.6**

^60^ Let $a = \left( {\mu ,\eta ,v} \right)$,$a_{1} = \left( {\mu_{1} ,\eta_{1} ,v_{1} } \right)$ and $a_{2} = \left( {\mu_{2} ,\eta_{2} ,v_{2} } \right)$ are three T-SFNs and $\lambda > 0$, then, some algebraic operations for T-SFNs are defined as follows:

1. 1 $$a_{1} \oplus a_{2} = \left( {\left( {1 - \left( {1 - \mu_{1}^{q} } \right)\left( {1 - \mu_{2}^{q} } \right)} \right)^{{{1/q}}} ,\eta_{1} \eta_{1} ,v_{1} v_{2} } \right)$$
2. 2 $$a_{1} \otimes a_{2} = \left( {\mu_{1} \mu_{2} ,\left( {1 - \left( {1 - \eta_{1}^{q} } \right)\left( {1 - \eta_{2}^{q} } \right)} \right)^{{{1 /q}}} ,\left( {1 - \left( {1 - v_{1}^{q} } \right)\left( {1 - v_{2}^{q} } \right)} \right)^{{{1/q}}} } \right)$$
3. 3 $$\lambda a = \left( {\left( {1 - \left( {1 - \mu^{q} } \right)^{\lambda } } \right)^{{{1 /q}}} ,\eta^{\lambda } ,v^{\lambda } } \right)$$
4. 4 $$a^{\lambda } = \left( {\mu^{\lambda } ,\left( {1 - \left( {1 - \eta^{q} } \right)^{\lambda } } \right)^{{{1/q}}} ,\left( {1 - \left( {1 - v^{q} } \right)^{\lambda } } \right)^{{{1/q}}} } \right)$$

### **Definition 2.7**

^45^ Suppose $a = \left( {\mu ,\eta ,\nu } \right)$ be a T-SFN. Then, the score function is defined as follows:

5 $$SCR\left( a \right) = \mu^{q} - v^{q}$$

where $SCR\left( a \right) \in \left[ { - 1,1} \right]$ and $q \ge 1$.and the accuracy function $ACR\left( a \right)$ is defined as follow:

$$ACR\left( a \right) = \mu^{q} + \eta^{q} + v^{q}$$

where $$ACR\left( a \right) \in \left[ {0,1} \right]$$ and $$q \ge 1$$.

Let us suppose that $a_{1}$ and $a_{2}$ be two T- SFNs. Then,

1. If $$SCR\left( {a_{1} } \right) > SCR\left( {a_{2} } \right),$$ then $$a_{1} > a_{2}$$,
2. If $$SCR\left( {a_{1} } \right) < SCR\left( {a_{2} } \right),$$$$a_{1} < a_{2}$$,
3. $$SCR\left( {a_{1} } \right) = SCR\left( {a_{2} } \right),$$ then
  i. If $$ACR\left( {a_{1} } \right) > ACR\left( {a_{2} } \right),$$ then $$a_{1} > a_{2}$$,
  ii. $$ACR\left( {a_{1} } \right) = ACR\left( {a_{2} } \right),$$ then $$a_{1} = a_{2}$$.

### **Definition 2.8**

^61^ Let us suppose that $r \ge 0$, $s \ge 0$ and $a_{k} \,;\,\,\,\,\left( {k = 1,2,...,n} \right)$ is a set of positive integers, the BM operator is defined as follows.

$$BM^{r,s} \left( {a_{1} ,a_{2} ,...,a_{n} } \right) = \left( {\frac{1}{{n\left( {n - 1} \right)}}\sum\limits_{\begin{subarray}{l} i,j = 1 \\ i \ne j \end{subarray} }^{n} {a_{i}^{s} a_{j}^{r} } } \right)^{{{1/{r + s}}}}$$

The BM operator has the capability to take into consideration the interaction between any two inputs. Depending on how these arguments relate to one another, it may be possible to divide input variables into a number of autonomous groups. These groups may engage with one another while remaining independent of one another.

### **Definition 2.9**

^62^ Suppose that $r \ge 0$, $s \ge 0$ and $a_{k} \,;\,\,\,\,\left( {k = 1,2,...,n} \right)$ is a set of positive integers, the PBM operator is defined as follows.

$$PBM^{r,s} \left( {a_{1} ,a_{2} ,...,a_{n} } \right) = \frac{1}{d}\left( {\sum\limits_{h = 1}^{d} {\left( {\frac{1}{{\left| {P_{h} } \right|}}\sum\limits_{{i \in P_{h} }} {a_{i}^{r} \left( {\frac{1}{{\left| {P_{h} } \right| - 1}}\sum\limits_{{j \in P_{h} }} {a_{j}^{s} } } \right)} } \right)^{{{1/{r + s}}}} } } \right)$$

where $\left| {P_{h} } \right|$ is the cardinality of $P_{h}$, $d$ is the number of independent groups and $\sum\limits_{h = 1}^{d} {\left| {P_{h} } \right|} = n$.

## T-spherical fuzzy partitioned Bonferroni mean operator

Considering the partitioned structure among criteria, we extend the PBM operators to accommodate the T-spherical fuzzy environment. Based on the results of definitions discussed above, we give the definition of T-spherical fuzzy partitioned Bonferroni mean operator and T-spherical fuzzy weighted partitioned Bonferroni mean operator in this section.

### **Definition 3.1**

Let $a_{k} = \left( {\mu_{k} ,\eta_{k} ,v_{k} } \right),\,\,\,\left( {k = 1,2,...,n} \right)$ be the set of T-SFNs, which is divided in to $d$ distinct groups $P_{1} ,P_{2} ,...,P_{d}$. For any $r,s \ge 0$, with $r + s > 0$ we give the definition of T-SFPBM operator as follows:

6 $$T{\text{-}}SFPBM^{r,s} \left( {a_{1} ,a_{2} ,...,a_{n} } \right) = \frac{1}{d}\left( {\sum\limits_{h = 1}^{d} {\left( {\frac{1}{{\left| {P_{h} } \right|}}\sum\limits_{{i \in P_{h} }} {a_{i}^{r} \left( {\frac{1}{{\left| {P_{h} } \right| - 1}}\sum\limits_{{j \in P_{h} }} {a_{j}^{s} } } \right)} } \right)^{{{1/{r + s}}}} } } \right)$$

where $\left| {P_{h} } \right|$ is the cardinality of $P_{h}$, $d$ is the number of independent groups and $\cup_{h = 1}^{d} \left| {P_{h} } \right| = n$.

### **Theorem 3.1**

*Suppose that* $a_{k} = \left( {\mu_{k} ,\eta_{k} ,v_{k} } \right),\,\,\,\left( {k = 1,2,...,n} \right)$ *be the set of T-SFNs, which is partitioned in to* $d$ *distinct classes* $P_{1} ,P_{2} ,...,P_{d}$. *Then, the aggregated value of* $T{\text{-}}SFPBM^{r,s}$ *is also a T-SFN, which has the following form:*

7 $$T{\text{-}}SFPBM^{r,s} \left( {a_{1} ,a_{2} ,...,a_{n} } \right) = \left( \begin{aligned} & \left( {1 - \prod\limits_{h = 1}^{d} {\left( {1 - \left( {1 - \prod\limits_{\begin{subarray}{l} i,j \in P_{h} \\ i \ne j \end{subarray} } {\left( {1 - \mu_{i}^{qr} \mu_{j}^{qs} } \right)^{{\frac{1}{{\left| {P_{h} } \right|\left( {\left| {P_{h} } \right| - 1} \right)}}}} } } \right)^{{\frac{1}{r + s}}} } \right)^{\frac{1}{d}} } } \right)^{\frac{1}{q}} , \hfill \\ & \left( {\prod\limits_{h = 1}^{d} {\left( {1 - \left( {1 - \prod\limits_{\begin{subarray}{l} i,j \in P_{h} \\ i \ne j \end{subarray} } {\left( {1 - \left( {1 - \eta_{i}^{q} } \right)^{r} \left( {1 - \eta_{j}^{q} } \right)^{s} } \right)^{{\frac{1}{{\left| {P_{h} } \right|\left( {\left| {P_{h} } \right| - 1} \right)}}}} } } \right)^{{\frac{1}{r + s}}} } \right)^{\frac{1}{d}} } } \right)^{\frac{1}{q}} , \hfill \\ & \left( {\prod\limits_{h = 1}^{d} {\left( {1 - \left( {1 - \prod\limits_{\begin{subarray}{l} i,j \in P_{h} \\ i \ne j \end{subarray} } {\left( {1 - \left( {1 - v_{i}^{q} } \right)^{r} \left( {1 - v_{j}^{q} } \right)^{s} } \right)^{{\frac{1}{{\left| {P_{h} } \right|\left( {\left| {P_{h} } \right| - 1} \right)}}}} } } \right)^{{\frac{1}{r + s}}} } \right)^{\frac{1}{d}} } } \right)^{\frac{1}{q}} \hfill \\ \end{aligned} \right)$$

### **Definition 3.2**

Let $a_{k} = \left( {\mu_{k} ,\eta_{k} ,v_{k} } \right),\,\,\,\left( {k = 1,2,...,n} \right)$ be the set of T-SFNs, which is partitioned in to $d$ distinct classes $P_{1} ,P_{2} ,...,P_{d}$. For any $r,s \ge 0$, with $r + s > 0$ we give the definition of T-SFWPBM operator as follows:

8 $$T{\text{-}}SFWPBM^{r,s} \left( {a_{1} ,a_{2} ,...,a_{n} } \right) = \frac{1}{d}\left( {\sum\limits_{h = 1}^{d} {\left( {\frac{1}{{\left| {P_{h} } \right|}}\sum\limits_{{i \in P_{h} }} {w_{i}^{r} a_{i}^{r} \left( {\frac{1}{{\left| {P_{h} } \right| - 1}}\sum\limits_{{j \in P_{h} }} {w_{j}^{s} a_{j}^{s} } } \right)} } \right)^{{{1 / {r + s}}}} } } \right)$$

where $\left| {P_{h} } \right|$ is the cardinality of $P_{h}$, $d$ is the number of independent groups and $\cup_{h = 1}^{d} \left| {P_{h} } \right| = n$. $w = \left( {w_{1} ,w_{2} ,...,w_{n} } \right)$ is the weight vector of $a_{k} = \left( {\mu_{k} ,\eta_{k} ,v_{k} } \right),\,\,\,\left( {k = 1,2,...,n} \right)$ satisfying $w_{j} \in \left[ {0,1} \right],\,\,\,j = 1,2,...,m$ and $\sum\limits_{j = 1}^{m} {w_{j} } = 1$.

### **Theorem 3.2**

*Suppose that* $a_{k} = \left( {\mu_{k} ,\eta_{k} ,v_{k} } \right),\,\,\,\left( {k = 1,2,...,n} \right)$ *be the set of T-SFNs, which is partitioned in to* $d$ *distinct classes* $P_{1} ,P_{2} ,...,P_{d}$*. Then, the aggregated value of* $T{\text{-}}SFWPBM^{r,s}$ *is also a T-SFN, which has the following form:*

9 $$\begin{gathered} T{\text{-}}SFWPBM^{r,s} \left( {a_{1} ,a_{2} ,...,a_{n} } \right) \hfill \\ = \left( \begin{gathered} \left( {1 - \prod\limits_{h = 1}^{d} {\left( {1 - \left( {1 - \prod\limits_{\begin{subarray}{l} i,j \in P_{h} \\ i \ne j \end{subarray} } {\left( {1 - \left( {1 - \left( {1 - \mu_{i}^{qr} } \right)^{{w_{i}^{r} }} } \right)\left( {1 - \left( {1 - \mu_{j}^{qs} } \right)^{{w_{j}^{s} }} } \right)} \right)^{{\frac{1}{{\left| {P_{h} } \right|\left( {\left| {P_{h} } \right| - 1} \right)}}}} } } \right)^{{\frac{1}{r + s}}} } \right)^{\frac{1}{d}} } } \right)^{\frac{1}{q}} , \hfill \\ \left( {1 - \prod\limits_{h = 1}^{d} {\left( {1 - \left( {1 - \prod\limits_{\begin{subarray}{l} i,j \in P_{h} \\ i \ne j \end{subarray} } {\left( {1 - \left( {1 - \left( {1 - \eta_{i}^{qr} } \right)^{{w_{i}^{r} }} } \right)\left( {1 - \left( {1 - \eta_{j}^{qs} } \right)^{{w_{j}^{s} }} } \right)} \right)^{{\frac{1}{{\left| {P_{h} } \right|\left( {\left| {P_{h} } \right| - 1} \right)}}}} } } \right)^{{\frac{1}{r + s}}} } \right)^{\frac{1}{d}} } } \right)^{\frac{1}{q}} , \hfill \\ \left( {1 - \prod\limits_{h = 1}^{d} {\left( {1 - \left( {1 - \prod\limits_{\begin{subarray}{l} i,j \in P_{h} \\ i \ne j \end{subarray} } {\left( {1 - \left( {1 - \left( {1 - v_{i}^{qr} } \right)^{{w_{i}^{r} }} } \right)\left( {1 - \left( {1 - v_{j}^{qs} } \right)^{{w_{j}^{s} }} } \right)} \right)^{{\frac{1}{{\left| {P_{h} } \right|\left( {\left| {P_{h} } \right| - 1} \right)}}}} } } \right)^{{\frac{1}{r + s}}} } \right)^{\frac{1}{d}} } } \right)^{\frac{1}{q}} \hfill \\ \end{gathered} \right) \hfill \\ \end{gathered}$$

### *Proof*

From Eq. (4) and Definition 2.6, we know that,

10 $$a_{i} = \left( {\mu_{i} ,\eta_{i} ,v_{i} } \right),\;\;a_{i}^{r} = \left( {\mu_{i}^{r} ,\left( {1 - \left( {1 - \eta_{i}^{q} } \right)^{r} } \right)^{{{1/q}}} ,\left( {1 - \left( {1 - v_{i}^{q} } \right)^{r} } \right)^{{{1/q}}} } \right),$$

11 $$a_{j} = \left( {\mu_{j} ,\eta_{j} ,v_{j} } \right),\;\;a_{j}^{s} = \left( {\mu_{j}^{s} ,\left( {1 - \left( {1 - \eta_{j}^{q} } \right)^{s} } \right)^{{{1/q}}} ,\left( {1 - \left( {1 - v_{j}^{q} } \right)^{s} } \right)^{{{1/q}}} } \right)$$

According to Eqs. (3), (10) and (11), we get,

12 $$w_{i}^{r} a_{i}^{r} = \left( {\left( {1 - \left( {1 - \mu_{i}^{qr} } \right)^{{w_{i}^{r} }} } \right)^{{{1/q}}} ,\left( {1 - \left( {1 - \eta_{i}^{q} } \right)^{r} } \right)^{{{{w_{i}^{r} } /q}}} ,\left( {1 - \left( {1 - v_{i}^{q} } \right)^{r} } \right)^{{{{w_{i}^{r} }/q}}} } \right)$$

13 $$w_{j}^{s} a_{j}^{s} = \left( {\left( {1 - \left( {1 - \mu_{j}^{qs} } \right)^{{w_{j}^{s} }} } \right)^{{{1/q}}} ,\left( {1 - \left( {1 - \eta_{j}^{q} } \right)^{s} } \right)^{{{{w_{j}^{s} } / q}}} ,\left( {1 - \left( {1 - v_{j}^{q} } \right)^{s} } \right)^{{{{w_{j}^{s} }/q}}} } \right)$$

Now, from Eqs. (12) and (13), we obtain

14 $$\left( {w_{i}^{r} a_{i}^{r} } \right)\left( {w_{j}^{s} a_{j}^{s} } \right) = \left( \begin{aligned} & \left( {1 - \left( {1 - \mu_{i}^{qr} } \right)^{{w_{i}^{r} }} } \right)^{{{1 /q}}} \left( {1 - \left( {1 - \mu_{j}^{qs} } \right)^{{w_{j}^{s} }} } \right)^{{{1 /q}}} ,\left( {1 - \left( {1 - \left( {1 - \left( {1 - \eta_{i}^{q} } \right)^{r} } \right)^{{w_{i}^{r} }} } \right)\left( {1 - \left( {1 - \left( {1 - \eta_{j}^{q} } \right)^{s} } \right)^{{w_{j}^{s} }} } \right)^{{{1/q}}} } \right), \hfill \\ & \left( {1 - \left( {1 - \left( {1 - \left( {1 - v_{i}^{q} } \right)^{r} } \right)^{{w_{i}^{r} }} } \right)\left( {1 - \left( {1 - \left( {1 - v_{j}^{q} } \right)^{s} } \right)^{{w_{j}^{s} }} } \right)^{{{1/q}}} } \right) \hfill \\ \end{aligned} \right)$$

Using mathematical induction on Eq. (14), we get

$$\begin{gathered} \sum\limits_{\begin{subarray}{l} i,j \in P_{h} \\ i \ne j \end{subarray} } {\left( {w_{i}^{r} a_{i}^{r} } \right)\left( {w_{j}^{s} a_{j}^{s} } \right)} \hfill \\ = \left( \begin{gathered} \left( {1 - \prod\limits_{\begin{subarray}{l} i,j \in P_{h} \\ i \ne j \end{subarray} } {\left( {1 - \left( {1 - \mu_{i}^{qr} } \right)^{{w_{i}^{r} }} } \right)\left( {1 - \left( {1 - \mu_{j}^{qs} } \right)^{{w_{j}^{s} }} } \right)} } \right)^{{{1 /q}}} ,\left( {\prod\limits_{\begin{subarray}{l} i,j \in P_{h} \\ i \ne j \end{subarray} } {\left( {1 - \left( {1 - \left( {1 - \left( {1 - \eta_{i}^{q} } \right)^{r} } \right)^{{w_{i}^{r} }} } \right)\left( {1 - \left( {1 - \left( {1 - \eta_{j}^{q} } \right)^{s} } \right)^{{w_{j}^{s} }} } \right)} \right)} } \right)^{{{1 / q}}} , \hfill \\ \left( {\prod\limits_{\begin{subarray}{l} i,j \in P_{h} \\ i \ne j \end{subarray} } {\left( {1 - \left( {1 - \left( {1 - \left( {1 - v_{i}^{q} } \right)^{r} } \right)^{{w_{i}^{r} }} } \right)\left( {1 - \left( {1 - \left( {1 - v_{j}^{q} } \right)^{s} } \right)^{{w_{j}^{s} }} } \right)} \right)} } \right)^{{{1 /q}}} \hfill \\ \end{gathered} \right) \hfill \\ \end{gathered}$$

Further,

$$\begin{gathered} \frac{1}{{\left| {P_{h} } \right|\left( {\left| {P_{h} } \right| - 1} \right)}}\sum\limits_{\begin{subarray}{l} i,j \in P_{h} \\ i \ne j \end{subarray} } {\left( {w_{i}^{r} a_{i}^{r} } \right)\left( {w_{j}^{s} a_{j}^{s} } \right)} \hfill \\ = \left( \begin{gathered} \left( {1 - \prod\limits_{\begin{subarray}{l} i,j \in P_{h} \\ i \ne j \end{subarray} } {\left( {1 - \left( {1 - \left( {1 - \mu_{i}^{qr} } \right)^{{w_{i}^{r} }} } \right)\left( {1 - \left( {1 - \mu_{j}^{qs} } \right)^{{w_{j}^{s} }} } \right)} \right)^{{\frac{1}{{\left| {P_{h} } \right|\left( {\left| {P_{h} } \right| - 1} \right)}}}} } } \right)^{{{1/q}}} ,\left( {\prod\limits_{\begin{subarray}{l} i,j \in P_{h} \\ i \ne j \end{subarray} } {\left( {1 - \left( {1 - \left( {1 - \left( {1 - \eta_{i}^{q} } \right)^{r} } \right)^{{w_{i}^{r} }} } \right)\left( {1 - \left( {1 - \left( {1 - \eta_{j}^{q} } \right)^{s} } \right)^{{w_{j}^{s} }} } \right)} \right)} } \right)^{{\frac{1}{q}.\frac{1}{{\left| {P_{h} } \right|\left( {\left| {P_{h} } \right| - 1} \right)}}}} , \hfill \\ \left( {\prod\limits_{\begin{subarray}{l} i,j \in P_{h} \\ i \ne j \end{subarray} } {\left( {1 - \left( {1 - \left( {1 - \left( {1 - v_{i}^{q} } \right)^{r} } \right)^{{w_{i}^{r} }} } \right)\left( {1 - \left( {1 - \left( {1 - v_{j}^{q} } \right)^{s} } \right)^{{w_{j}^{s} }} } \right)} \right)} } \right)^{{\frac{1}{q}.\frac{1}{{\left| {P_{h} } \right|\left( {\left| {P_{h} } \right| - 1} \right)}}}} \hfill \\ \end{gathered} \right) \hfill \\ \end{gathered}$$

Similarly,

$$\begin{gathered} \left( {\frac{1}{{\left| {P_{h} } \right|\left( {\left| {P_{h} } \right| - 1} \right)}}\sum\limits_{\begin{subarray}{l} i,j \in P_{h} \\ i \ne j \end{subarray} } {\left( {w_{i}^{r} a_{i}^{r} } \right)\left( {w_{j}^{s} a_{j}^{s} } \right)} } \right)^{{\frac{1}{r + s}}} \hfill \\ = \left( \begin{gathered} \left( {1 - \prod\limits_{\begin{subarray}{l} i,j \in P_{h} \\ i \ne j \end{subarray} } {\left( {1 - \left( {1 - \left( {1 - \mu_{i}^{qr} } \right)^{{w_{i}^{r} }} } \right)\left( {1 - \left( {1 - \mu_{j}^{qs} } \right)^{{w_{j}^{s} }} } \right)} \right)^{{\frac{1}{{\left| {P_{h} } \right|\left( {\left| {P_{h} } \right| - 1} \right)}}}} } } \right)^{{\frac{1}{q}.\frac{1}{r + s}}} , \hfill \\ \left( {1 - \left( {1 - \prod\limits_{\begin{subarray}{l} i,j \in P_{h} \\ i \ne j \end{subarray} } {\left( {1 - \left( {1 - \left( {1 - \left( {1 - \eta_{i}^{q} } \right)^{r} } \right)^{{w_{i}^{r} }} \left( {1 - \left( {1 - \left( {1 - \eta_{j}^{q} } \right)^{s} } \right)^{{w_{j}^{s} }} } \right)} \right)} \right)^{{\frac{1}{{\left| {P_{h} } \right|\left( {\left| {P_{h} } \right| - 1} \right)}}}} } } \right)^{{\frac{1}{r + s}}} } \right)^{{^{{{1/q}}} }} , \hfill \\ \left( {1 - \left( {1 - \prod\limits_{\begin{subarray}{l} i,j \in P_{h} \\ i \ne j \end{subarray} } {\left( {1 - \left( {1 - \left( {1 - \left( {1 - v_{i}^{q} } \right)^{r} } \right)^{{w_{i}^{r} }} \left( {1 - \left( {1 - \left( {1 - v_{j}^{q} } \right)^{s} } \right)^{{w_{j}^{s} }} } \right)} \right)} \right)^{{\frac{1}{{\left| {P_{h} } \right|\left( {\left| {P_{h} } \right| - 1} \right)}}}} } } \right)^{{\frac{1}{r + s}}} } \right)^{{^{{{1/q}}} }} \hfill \\ \end{gathered} \right) \hfill \\ \end{gathered}$$

By mathematical induction, we can get

$$\begin{gathered} \sum\limits_{h = 1}^{d} {\left( {\frac{1}{{\left| {P_{h} } \right|\left( {\left| {P_{h} } \right| - 1} \right)}}\sum\limits_{\begin{subarray}{l} i,j \in P_{h} \\ i \ne j \end{subarray} } {\left( {w_{i}^{r} a_{i}^{r} } \right)\left( {w_{j}^{s} a_{j}^{s} } \right)} } \right)^{{\frac{1}{r + s}}} } \hfill \\ = \left( \begin{gathered} \left( {1 - \prod\limits_{h = 1}^{d} {\left( {1 - \left( {1 - \prod\limits_{\begin{subarray}{l} i,j \in P_{h} \\ i \ne j \end{subarray} } {\left( {1 - \left( {1 - \left( {1 - \mu_{i}^{qr} } \right)^{{w_{i}^{r} }} } \right)\left( {1 - \left( {1 - \mu_{j}^{qs} } \right)^{{w_{j}^{s} }} } \right)} \right)^{{\frac{1}{{\left| {P_{h} } \right|\left( {\left| {P_{h} } \right| - 1} \right)}}}} } } \right)^{{\frac{1}{r + s}}} } \right)} } \right)^{{{1/q}}} , \hfill \\ \left( {\prod\limits_{h = 1}^{d} {\left( {1 - \left( {1 - \prod\limits_{\begin{subarray}{l} i,j \in P_{h} \\ i \ne j \end{subarray} } {\left( {1 - \left( {1 - \left( {1 - \left( {1 - \eta_{i}^{q} } \right)^{r} } \right)^{{w_{i}^{r} }} \left( {1 - \left( {1 - \left( {1 - \eta_{j}^{q} } \right)^{s} } \right)^{{w_{j}^{s} }} } \right)} \right)} \right)^{{\frac{1}{{\left| {P_{h} } \right|\left( {\left| {P_{h} } \right| - 1} \right)}}}} } } \right)^{{\frac{1}{r + s}}} } \right)} } \right)^{{{1 /q}}} , \hfill \\ \left( {\prod\limits_{h = 1}^{d} {\left( {1 - \left( {1 - \prod\limits_{\begin{subarray}{l} i,j \in P_{h} \\ i \ne j \end{subarray} } {\left( {1 - \left( {1 - \left( {1 - \left( {1 - v_{i}^{q} } \right)^{r} } \right)^{{w_{i}^{r} }} \left( {1 - \left( {1 - \left( {1 - v_{j}^{q} } \right)^{s} } \right)^{{w_{j}^{s} }} } \right)} \right)} \right)^{{\frac{1}{{\left| {P_{h} } \right|\left( {\left| {P_{h} } \right| - 1} \right)}}}} } } \right)^{{\frac{1}{r + s}}} } \right)} } \right)^{{{1/q}}} \hfill \\ \end{gathered} \right) \hfill \\ \end{gathered}$$

According to scaler multiplication property, we can obtain

$$\begin{gathered} T{\text{-}}SFWPBM^{r,s} \left( {a_{1} ,a_{2} ,...,a_{n} } \right) = \frac{1}{d}\left( {\sum\limits_{h = 1}^{d} {\left( {\frac{1}{{\left| {P_{h} } \right|}}\sum\limits_{{i \in P_{h} }} {w_{i}^{r} a_{i}^{r} \left( {\frac{1}{{\left| {P_{h} } \right| - 1}}\sum\limits_{{j \in P_{h} }} {w_{j}^{s} a_{j}^{s} } } \right)} } \right)^{{{1 / {r + s}}}} } } \right) \hfill \\ = \left( \begin{gathered} \left( {1 - \prod\limits_{h = 1}^{d} {\left( {1 - \left( {1 - \prod\limits_{\begin{subarray}{l} i,j \in P_{h} \\ i \ne j \end{subarray} } {\left( {1 - \left( {1 - \left( {1 - \mu_{i}^{qr} } \right)^{{w_{i}^{r} }} } \right)\left( {1 - \left( {1 - \mu_{j}^{qs} } \right)^{{w_{j}^{s} }} } \right)} \right)^{{\frac{1}{{\left| {P_{h} } \right|\left( {\left| {P_{h} } \right| - 1} \right)}}}} } } \right)^{{\frac{1}{r + s}}} } \right)^{\frac{1}{d}} } } \right)^{\frac{1}{q}} , \hfill \\ \left( {1 - \prod\limits_{h = 1}^{d} {\left( {1 - \left( {1 - \prod\limits_{\begin{subarray}{l} i,j \in P_{h} \\ i \ne j \end{subarray} } {\left( {1 - \left( {1 - \left( {1 - \eta_{i}^{qr} } \right)^{{w_{i}^{r} }} } \right)\left( {1 - \left( {1 - \eta_{j}^{qs} } \right)^{{w_{j}^{s} }} } \right)} \right)^{{\frac{1}{{\left| {P_{h} } \right|\left( {\left| {P_{h} } \right| - 1} \right)}}}} } } \right)^{{\frac{1}{r + s}}} } \right)^{\frac{1}{d}} } } \right)^{\frac{1}{q}} , \hfill \\ \left( {1 - \prod\limits_{h = 1}^{d} {\left( {1 - \left( {1 - \prod\limits_{\begin{subarray}{l} i,j \in P_{h} \\ i \ne j \end{subarray} } {\left( {1 - \left( {1 - \left( {1 - v_{i}^{qr} } \right)^{{w_{i}^{r} }} } \right)\left( {1 - \left( {1 - v_{j}^{qs} } \right)^{{w_{j}^{s} }} } \right)} \right)^{{\frac{1}{{\left| {P_{h} } \right|\left( {\left| {P_{h} } \right| - 1} \right)}}}} } } \right)^{{\frac{1}{r + s}}} } \right)^{\frac{1}{d}} } } \right)^{\frac{1}{q}} \hfill \\ \end{gathered} \right) \hfill \\ \end{gathered}$$

which completes the proof of Theorem 3.2.

### **Property 3.1**

(Idempotency) *If any T-SFN Suppose that* $a_{k} = \left( {\mu_{k} ,\eta_{k} ,v_{k} } \right),\,\,\,\left( {k = 1,2,...,n} \right)$ *are equal, i.e.* $a_{k} = a = \left( {\mu ,\eta ,v} \right)$ *, then* $T{\text{-}}SFPBM^{r,s} \left( {a_{1} ,a_{2} ,...,a_{n} } \right) = a$.

### **Property 3.2**

(Commutativity) *If* $a_{k}^{\prime}$ *is any permutation of* $a_{k} \,\left( {k = 1,2,...,n} \right)$*, then we have the relationship of the form:*

$$T{\text{-}}SFPBM^{r,s} \left( {a_{1} ,a_{2} ,...,a_{n} } \right) = T{\text{-}}SFPBM^{r,s} \left( {a_{1}^{\prime} ,a_{2}^{\prime} ,...,a_{n}^{\prime} } \right).$$

### **Property 3.3**

(Monotonicity) *Let* $a_{k} = \left( {\mu_{k} ,\eta_{k} ,v_{k} } \right),\,\,\,\left( {k = 1,2,...,n} \right)$ *and* $B_{k} = \left( {\mu_{k} ,\eta_{k} ,v_{k} } \right),\,$ $\left( {k = 1,2,...,n} \right)$ *be two collections of T-SFNs. If* $\mu_{{a_{k} }} \ge \mu_{{B_{k} }}$, $\eta_{{a_{k} }} \le \eta_{{B_{k} }}$ *and* $v_{{a_{k} }} \le v_{{B_{k} }}$ *then,*

$$T{\text{-}}SFPBM^{r,s} \left( {a_{1} ,a_{2} ,...,a_{n} } \right) \ge T{\text{-}}SFPBM^{r,s} \left( {B_{1} ,B_{2} ,...,B_{n} } \right)$$

### **Definition 3.3**

Let $a_{1} = \left( {\mu_{1} ,\eta_{1} ,v_{1} } \right)$ and $a_{2} = \left( {\mu_{2} ,\eta_{2} ,v_{2} } \right)$ be two TSFNs. Then, the Hamming distance ‘$d$’ between two T-SFNs is defined as follows,

15 $$d\left( {a_{1} ,a_{2} } \right) = \left( {\frac{1}{2}\left[ {\left| {\mu_{1}^{q} - \mu_{2}^{q} } \right| + \left| {\eta_{1}^{q} - \eta_{2}^{q} } \right| + \left| {v_{1}^{q} - v_{2}^{q} } \right|} \right]} \right)^{{{1 /q}}}$$

where the Hamming distance ‘$d$’ satisfies the following condition; $0 \le d\left( {a_{1} ,a_{2} } \right) \le 1$.

## Group decision-making framework based on IFM and aggregation operators for T-SFN

**Figure 1:**
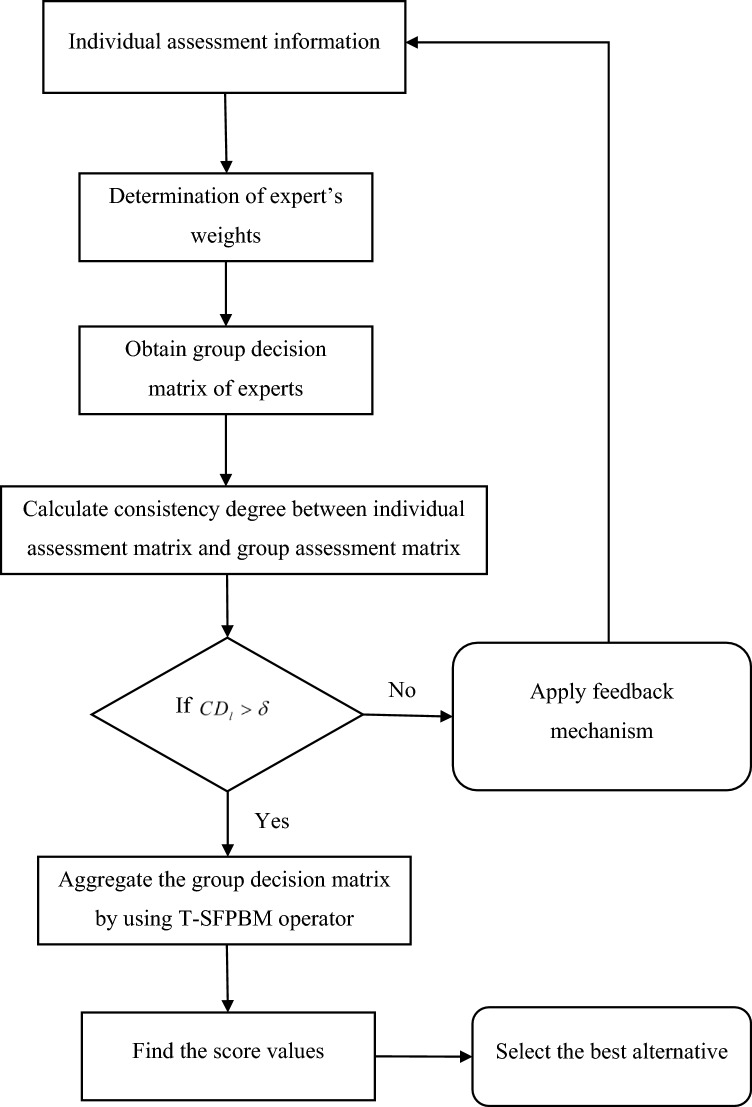
Framework of the proposed approach.

Group decision‐making usually involves two or more experts while solving any decision problem. However, due to different experts have distinct social experiences and personal beliefs, their decisions frequently reflect prejudice. Therefore, it might be challenging for specialists to agree upon a course of action in group decision-making. Experts' evaluation of any object can be expressed in terms of T-SFN. In this section, an IFM using T-SF information is proposed to assist experts to obtain consistency while dealing with any group decision-making problem. The flowchart of the decision-making process is shown in Fig. 1.

### Problem description

Let us consider that $X = \left\{ {x_{1} ,x_{2} ,...,x_{m} } \right\}$ be ‘m’ alternatives and $G = \left\{ {G_{1} ,G_{2} ,...,G_{n} } \right\}$ are ‘n’ attributes with weighting vector $w = \left( {w_{1} ,w_{2} ,...,w_{n} } \right)^{T}$ satisfying the condition that $\sum\nolimits_{j = 1}^{n} {w_{j} } = 1$ and $0 \le w_{j} \le 1$. Suppose that the ‘L’ experts $\left\{ {E^{\left( 1 \right)} ,E^{\left( 2 \right)} ,...,E^{\left( L \right)} } \right\}$ evaluated $X_{i} \left( {i = 1,2,...,m} \right)$ alternatives based on $G_{j} \left( {j = 1,2,...,n} \right)$ attributes and gave the assessment information in the form of T-SFN, i.e. $R^{\left( l \right)} = \left( {a_{ij}^{\left( l \right)} } \right)_{m \times n} ;\,l = 1,2,...,L$. Where $a_{ij}^{\left( l \right)} = \left( {\mu_{ij}^{\left( l \right)} ,\eta_{ij}^{\left( l \right)} ,v_{ij}^{\left( l \right)} } \right)$. To find the best alternative in group decision-making IFM-based technique is designed using T-SF setting. The computing steps are simply executed as follows:

**Step 1.**: Get T-SF decision matrices concerning experts’ opinions.
  16 $$R^{\left( l \right)} = \left( {a_{ij}^{\left( l \right)} } \right)_{m \times n} ;\,l = 1,2,...,L$$
  where $$a_{ij}^{\left( l \right)} = \left( {\mu_{ij}^{\left( l \right)} ,\eta_{ij}^{\left( l \right)} ,v_{ij}^{\left( l \right)} } \right)$$.
**Step 2.**: *(Determination of weights of experts)* To determine the weight of experts, the following procedure is given:
  **Step 2.1.**: First, we find the consistency degree between the evaluation matrices provided by experts; Let $R^{\left( 1 \right)} = \left( {a_{ij}^{\left( 1 \right)} } \right)_{m \times n}$ and $R^{\left( 2 \right)} = \left( {a_{ij}^{\left( 2 \right)} } \right)_{m \times n}$ be any two T-SF preference relation matrices. Then, the consistency degree between $R^{\left( 1 \right)}$ and $R^{\left( 2 \right)}$ is defined as follows^63^:
    17 $$C\left( {R^{\left( 1 \right)} ,R^{\left( 2 \right)} } \right) = 1 - \frac{1}{mn}\sum\limits_{i = 1}^{m} {\sum\limits_{j = 1}^{n} {d\left( {a_{ij}^{\left( 1 \right)} ,a_{ij}^{\left( 2 \right)} } \right)} }$$
    here ‘$d$’ is Hamming distance.
  **Step 2.2.**: By using the definition of consistency degree, the overall consistency level of experts $E^{\left( L \right)}$ is defined as follows^63^;
    18 $$CC_{l} = \sum\limits_{\begin{subarray}{l} t = 1, \\ t \ne l \end{subarray} }^{L} {C\left( {R^{\left( l \right)} ,R^{\left( t \right)} } \right)}$$
  **Step 2.3.**: ^63^ The weights of experts are calculated with the following equation,
    19 $$w_{l} = \frac{{CC_{l} }}{{\sum\nolimits_{l = 1}^{L} {CC_{l} } }}$$
**Step 3.**: Aggregate the matrix $R^{\left( l \right)} = \left( {a_{ij}^{\left( l \right)} } \right)_{m \times n} ;\,l = 1,2,...,L$ of each expert to get the T-SF group decision matrix by utilizing the weights of experts, which can be computed as follows^63^;
  20 $$M_{1} = \sum\limits_{l = 1}^{L} {w_{l} \,} a_{ij}^{\left( l \right)}$$
**Step 4.**: Normalize the group T-SF decision matrix. If the criterion is benefit type, then do nothing; if the criterion is cost type, then the cost type criterion should be converted into a benefit type. The normalized group decision matrix is shown as follow;
  21 $$M_{1} = a_{ij} = \left( {\mu_{ij} ,\eta_{ij} ,v_{ij} } \right) = \left\{ \begin{aligned} & \left( {\mu_{ij} ,\eta_{ij} ,v_{ij} } \right),\,\,\,\,for\,\,benefit\,\,type\,\,G_{j} \hfill \\ & \left( {v_{ij} ,\eta_{ij} ,\mu_{ij} } \right),\,\,\,for\,\,\cos t\,\,type\,\,G_{j} \hfill \\ \end{aligned} \right.$$
**Step 5.**: To compute the consistency degree $CD_{l} \,\left( {l = 1,2,...,L} \right)$ between the evaluation decision matrices and group decision matrix, we utilize the Eq. (15) as follows;
  22 $$CD_{l} = C\left( {R^{\left( l \right)} ,M_{1} } \right) = 1 - \frac{1}{mn}\sum\limits_{i = 1}^{m} {\sum\limits_{j = 1}^{n} {d\left( {a_{ij}^{\left( l \right)} ,a_{ij} } \right)} }$$
  Obviously, $CD_{l} \in \left[ {0,1} \right]$. The larger the values of $CD_{l}$, the higher the consensus among experts. In general, achieving a unanimous agreement among experts is impossible. Therefore, a soft consensus is adopted in CRP, and a consensus threshold $\zeta \in \left[ {0,1} \right]$ is predefined to measure the consistency degree among experts.
**Step 6.**: If $CD_{l} > \delta$, then a consensus is achieved and we will move directly to step 8. Otherwise, the feedback process is carried out to promote the resulting consensus.
**Step 7.**: *(Feedback mechanism)* If $CD_{l}$ is less than the predefined consensus threshold, then by taking the evaluation matrix of an expert with the highest consistency degree as a reference, the expert with the lowest consistency degree will adjust his assessment value to a new decision matrix $R_{\varsigma }^{\left( l \right)} = \left( {a_{ij}^{\left( l \right)} } \right)_{m \times n} ;\,\left( {\varsigma = 1,2,...} \right)$ and go back to step 2, to recalculate the weights of experts and reapply all the procedure to compute the $CD_{l}$.
**Step 8.**: When the predefined threshold is less than the $CD_{l}$, then by applying, $T{\text{-}}SFWPBM^{r,s}$ we get the aggregated decision matrix; by Eq. (9).
**Step 9.**: The score function is calculated by using Eq. (5), to get the best alternatives among all of them. The higher values of score function will be the best one alternative.

## Numerical example and comparative analysis

This section aims to demonstrate the usability and efficiency of the technique suggested above by providing a numerical example of supplier selection for emergency medical supplies. Further, the impact of parameters on decision results and comparison analysis with existing methods is also given in the same section.

### Numerical example

Emergency medical supplies (EMS) are a collection of essential medical items that are prepared and kept in advance for use during times of crisis or emergency. These supplies are typically intended to provide medical care to individuals who have suffered injuries, illnesses, or other medical emergencies during a natural disaster, accident, or other unforeseen events that could disrupt access to medical services.

Some common examples of emergency medical supplies include:

- First aid kit with necessary medical supplies such as bandages, gauze, antiseptics, and pain relievers
- Automatic external defibrillator (AED) for cardiac emergencies
- Oxygen tanks and masks for respiratory emergencies
- Splints and braces for broken bones or fractures
- Tourniquets and wound dressings for heavy bleeding
- Medications such as epinephrine for allergic reactions or naloxone for opioid overdoses
- Portable suction units for airway management
- Intravenous (IV) fluids and supplies for dehydration or shock.

The specific items that makeup emergency medical supplies may vary depending on the nature of the emergency, the location, and the level of medical expertise available. However, the goal of emergency medical supplies is to provide medical care to individuals who have suffered injuries or illnesses until they can receive proper medical treatment.

**Figure 2:**
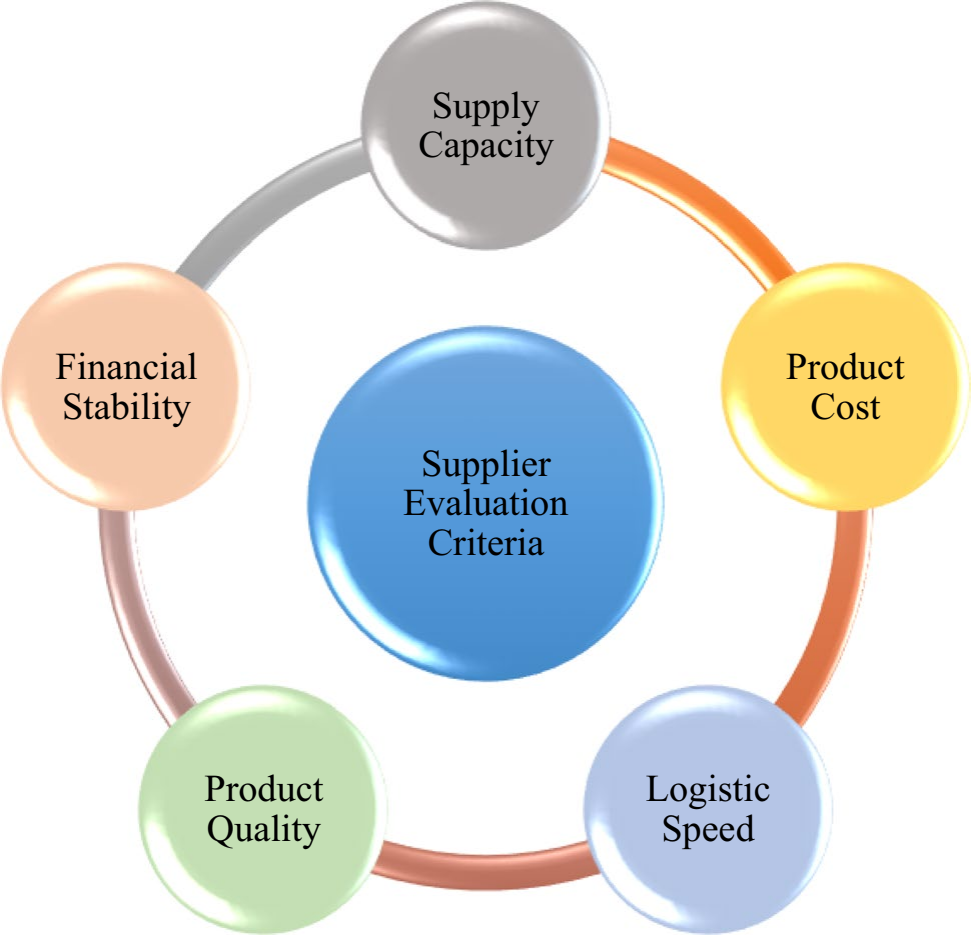
Criteria of supplier evaluation for emergency medical supplies.

The need for EMS has increased in recent years due to the regular occurrence of disasters, including epidemics, landslides, and earthquakes. We know that the need for medical protective equipment has increased since the COVID-19 epidemic, according to a survey conducted by the World Health Organization. For dealing with natural disasters, public health emergencies, and public safety events, emergency supplies are essential. The availability of emergency medical supplies in the case of a public emergency is crucial for ensuring social stability, protecting people's lives, and providing a considerable level of assurance for preventing the spread of disasters. How to rapidly and efficiently choose emergency supplies suppliers has significant practical significance for the timely supply of emergency medical supplies in light of the ambiguity, complexity, and fuzziness of the information environment. It is also an important issue for all sectors to deal with new confronts and construct an up-to-date emergency support model. Now, we suppose that there are three experts $\left( {D^{1} ,D^{2} ,D^{3} } \right)$ assess the four alternatives of emergency medical supplies suppliers, which are $\left( {x_{1} ,x_{2} ,x_{3} ,x_{4} } \right)$. The Five attributes (Fig. 2), which are effective in prioritizing these alternatives are as follows:

- **Supply capacity** $\left( {G_{1} } \right)$: Supply capacity is the promise made by suppliers to always have enough capacity to produce goods as agreed with enterprises.
- **Product cost** $\left( {G_{2} } \right)$: The cost of medical supplies is a significant factor in supplier selection. Consider the supplier's pricing, discounts, and payment terms, as well as the total cost of ownership.
- **Logistic speed** $\left( {G_{3} } \right)$: The speed at which emergency medical supplies are delivered by a supplier is a crucial factor to consider when selecting a supplier, particularly during crisis situations where time is of the essence. To evaluate a supplier's logistic speed for emergency medical supplies, consider factors such as their delivery time, inventory management practices, distribution network, tracking and communication capabilities, and emergency response capabilities. By assessing these factors, healthcare facilities can choose a supplier that has the necessary speed and capabilities to quickly deliver high-quality emergency medical supplies during emergency situations.
- **Product quality** $\left( {G_{4} } \right)$**:** The quality of medical supplies is essential for patient safety and healthcare outcomes. Consider the supplier's reputation, certifications, and quality assurance procedures, as well as product testing and validation.
- **Financial stability** $\left( {G_{5} } \right)$: Selecting a financially stable supplier for emergency medical supplies is crucial to ensure a reliable supply of essential products, particularly during crisis situations. To evaluate the financial stability of potential suppliers, consider their payment terms, creditworthiness, insurance coverage, contract terms, and business continuity planning. By assessing these factors, healthcare facilities can choose a supplier that has a stable financial foundation, reducing the risk of supply chain disruptions during emergency situations.

By carefully evaluating suppliers based on these factors, healthcare facilities can select high-quality suppliers that can provide reliable and cost-effective medical supplies even during times of crisis or disaster. Furthermore, emergency management involves a systematic approach to prevent, prepare for, respond to, and recover from disasters or emergency situations. In this paper, the phases of emergency management are divided into four sections which we presented as ‘$d$’ are as follows:

- *Mitigation (1):* It involves facility protection systems implementation, network architecture, resource allocation, and vital supply routes. It also covers the placement of early warning systems and help facilities. Buying flood and fire insurance for your home is a mitigation activity. Mitigation activities take place before and after emergencies.
- *Preparedness (2):* The preparedness phase is an integral part of the emergency management cycle and is aimed at enhancing the capacity and readiness of individuals, communities, and organizations to respond to an emergency or disaster. Its primary objective is to mitigate the impact of such situations by proactively preparing to manage and respond to them. During the preparedness phase, various activities are carried out, including emergency planning, training and education, resource management, and conducting exercises and drills. Emergency planning involves identifying potential hazards and risks, determining roles and responsibilities, and establishing procedures for communication and decision-making. Training and education are provided to emergency responders, community members, and public officials to equip them with the necessary knowledge and skills for effective response. Resource management strategies are developed to manage personnel, equipment, and supplies required during an emergency response. Exercises and drills are conducted to test emergency plans, identify areas for improvement, and enhance readiness. Overall, the preparedness phase ensures that individuals, communities, and organizations are prepared and equipped to respond to emergencies or disasters, thereby minimizing their impact and saving lives. Effective preparedness efforts are critical for reducing the impact of emergencies or disasters.
- *Response (3):* In certain circumstances, it starts when early warning systems or warnings from risks monitoring alert, the authorities of an impending disaster. Overall, the goal of the response phase is to stabilize the situation, protect lives and property, and begin the process of recovery. Successful response efforts are critical to minimizing the impact of a disaster and ultimately reducing the overall cost of the disaster.
- *Recovery (4):* The recovery phase is the final phase in the emergency management cycle and begins after the emergency or disaster has been brought under control. Its main goal is to restore the community and its infrastructure to its pre-disaster state or a new, improved state. The recovery phase typically involves activities such as assessing the damage caused by the disaster, cleaning up and restoring damaged infrastructure and property, supporting the psychological well-being of those affected by the disaster, and rebuilding the affected community to be more resilient to future disasters. Recovery efforts may take a considerable amount of time and require coordination among various organizations such as government agencies, non-governmental organizations, businesses, and community groups.

The nature of the emergency itself dictates these choices and actions, which presents a unique set of obstacles and challenges when creating or constructing management support systems. By following a structured approach to emergency management that encompasses all four phases, organizations can help mitigate the impact of disasters or emergencies, protect lives and property, and ensure that the affected communities recover as quickly as possible. Here we take $$q = 3$$, $$r = 1,$$$$s = 1$$ and five attributes that are independent of each other whose weighting vector is $$\left( {0.20,0.35,0.10,0.24,0.11} \right)$$, and the consistency threshold is $$\delta = 0.72$$. The evaluation information provided by three experts $$\left( {D^{1} ,D^{2} ,D^{3} } \right)$$ related to these four alternatives $$\left( {x_{1} ,x_{2} ,x_{3} ,x_{4} } \right)$$ based on the attributes $$\left( {G_{1} ,G_{2} ,G_{3} ,G_{4} ,G_{5} } \right)$$ is given in the form of T-SFNs shown in Tables 1, 2, 3. The proposed method is applied to select the best supplier for EMS and the concrete steps are as follows.

**Table 1:** Assessment matrix $R^{\left( 1 \right)} = \left( {a_{ij}^{\left( 1 \right)} } \right)_{4 \times 5}$.

	$G_{1}$	$G_{2}$	$G_{3}$	$G_{4}$	$G_{5}$
$x_{1}$	$\left( {0.5,0.4,0.2} \right)$	$\left( {0.4,0.6,0.7} \right)$	$\left( {0.5,0.1,0.4} \right)$	$\left( {0.6,0.2,0.3} \right)$	$\left( {0.1,0.7,0.4} \right)$
$x_{2}$	$\left( {0.3,0.5,0.8} \right)$	$\left( {0.4,0.3,0.2} \right)$	$\left( {0.8,0.3,0.2} \right)$	$\left( {0.6,0.4,0.7} \right)$	$\left( {0.5,0.2,0.6} \right)$
$x_{3}$	$\left( {0.6,0.5,0.3} \right)$	$\left( {0.3,0.4,0.8} \right)$	$\left( {0.5,0.4,0.9} \right)$	$\left( {0.5,0.1,0.4} \right)$	$\left( {0.6,0.5,0.1} \right)$
$x_{4}$	$\left( {0.4,0.4,0.6} \right)$	$\left( {0.7,0.2,0.5} \right)$	$\left( {0.8,0.1,0.5} \right)$	$\left( {0.4,0.3,0.6} \right)$	$\left( {0.8,0.1,0.7} \right)$

**Table 2:** Assessment matrix $R^{\left( 2 \right)} = \left( {a_{ij}^{\left( 2 \right)} } \right)_{4 \times 5}$.

	$G_{1}$	$G_{2}$	$G_{3}$	$G_{4}$	$G_{5}$
$x_{1}$	$\left( {0.3,0.1,0.9} \right)$	$\left( {0.6,0.1,0.7} \right)$	$\left( {0.5,0.3,0.5} \right)$	$\left( {0.3,0.1,0.7} \right)$	$\left( {0.8,0.1,0.5} \right)$
$x_{2}$	$\left( {0.8,0.2,0.1} \right)$	$\left( {0.5,0.4,0.3} \right)$	$\left( {0.4,0.3,0.6} \right)$	$\left( {0.7,0.6,0.2} \right)$	$\left( {0.2,0.6,0.4} \right)$
$x_{3}$	$\left( {0.7,0.2,0.6} \right)$	$\left( {0.7,0.2,0.5} \right)$	$\left( {0.5,0.7,0.2} \right)$	$\left( {0.3,0.8,0.1} \right)$	$\left( {0.6,0.8,0.1} \right)$
$x_{4}$	$\left( {0.8,0.1,0.7} \right)$	$\left( {0.5,0.4,0.6} \right)$	$\left( {0.4,0.3,0.6} \right)$	$\left( {0.1,0.7,0.3} \right)$	$\left( {0.7,0.1,0.4} \right)$

**Table 3:** Assessment matrix $R^{\left( 3 \right)} = \left( {a_{ij}^{\left( 3 \right)} } \right)_{4 \times 5}$.

	$G_{1}$	$G_{2}$	$G_{3}$	$G_{4}$	$G_{5}$
$x_{1}$	$\left( {0.7,0.3,0.2} \right)$	$\left( {0.8,0.1,0.5} \right)$	$\left( {0.6,0.4,0.5} \right)$	$\left( {0.2,0.3,0.7} \right)$	$\left( {0.8,0.2,0.6} \right)$
$x_{2}$	$\left( {0.3,0.8,0.1} \right)$	$\left( {0.9,0.2,0.3} \right)$	$\left( {0.5,0.2,0.4} \right)$	$\left( {0.7,0.2,0.5} \right)$	$\left( {0.8,0.2,0.1} \right)$
$x_{3}$	$\left( {0.4,0.3,0.6} \right)$	$\left( {0.7,0.5,0.1} \right)$	$\left( {0.6,0.2,0.7} \right)$	$\left( {0.3,0.7,0.5} \right)$	$\left( {0.5,0.1,0.4} \right)$
$x_{4}$	$\left( {0.6,0.3,0.7} \right)$	$\left( {0.7,0.6,0.2} \right)$	$\left( {0.8,0.1,0.7} \right)$	$\left( {0.4,0.1,0.5} \right)$	$\left( {0.4,0.3,0.8} \right)$

**Step 1.**: Construct the T-SF decision matrices according to the Eq. (16).
**Step 2.**: The weights of experts are computed as follows:
  **Step 2.1**: By using Eqs. (15), (16), and (17), the consistency degree between the evaluation matrices is obtained,
    $$C\left( {R^{\left( 1 \right)} ,R^{\left( 2 \right)} } \right) = 0.5934$$, $$C\left( {R^{\left( 1 \right)} ,R^{\left( 3 \right)} } \right) = 0.5643$$, $$C\left( {R^{\left( 2 \right)} ,R^{\left( 3 \right)} } \right) = 0.6169$$.
  **Step 2.2**: By using Eq. (18), the overall consistency degree is computed, which is given as follows:
    $$CC_{1} = 1.1577$$ , $$CC_{2} = 1.2103$$, $$CC_{3} = 1.1812$$.
  **Step 2.3**: By using Eq. (19), the weights of expert are obtained as follows:$w_{1} = \frac{{CC_{1} }}{{CC_{1} + CC_{2} + CC_{3} }} = 0.3261$, $w_{2} = \frac{{CC_{2} }}{{CC_{1} + CC_{2} + CC_{3} }} = 0.3410$,
    $$w_{3} = \frac{{CC_{3} }}{{CC_{1} + CC_{2} + CC_{3} }} = 0.3328$$
**Step 3.**: By utilizing weights of experts and Eqs. (1), (4) and (20), the group decision matrix $M_{1} = \left( {a_{ij} } \right)_{4 \times 5}$ is obtained. For Example
  $$a_{11} = \sum\limits_{l = 1}^{L} {w_{l} \,} a_{ij}^{\left( l \right)} = w_{1} a_{11}^{\left( 1 \right)} + w_{2} a_{11}^{\left( 2 \right)} + w_{3} a_{11}^{\left( 3 \right)}$$
  $$a_{12} = \sum\limits_{l = 1}^{L} {w_{l} \,} a_{ij}^{\left( l \right)} = w_{1} a_{12}^{\left( 1 \right)} + w_{2} a_{12}^{\left( 2 \right)} + w_{3} a_{12}^{\left( 3 \right)}$$
  Similarly, we get other $a_{ij} ,$ $i = 1,2,3,4$ and $j = 1,2,3,4,5$. The group decision matrix $M_{1} = \left( {a_{ij} } \right)_{4 \times 5} ;$ is shown in Table 4.

**Table 4:** Group decision matrix $M_{1} = \left( {a_{ij} } \right)_{4 \times 5}$.

	$G_{1}$	$G_{2}$	$G_{2}$	$G_{4}$	$G_{5}$
$x_{1}$	$\left( {0.65,0.23,0.84} \right)$	$\left( {0.75,0.42,0.36} \right)$	$\left( {0.64,0.38,0.45} \right)$	$\left( {0.43,0.27,0.59} \right)$	$\left( {0.84,0.41,0.57} \right)$
$x_{2}$	$\left( {0.72,0.45,0.68} \right)$	$\left( {0.68,0.22,0.18} \right)$	$\left( {0.82,0.15,0.47} \right)$	$\left( {0.59,0.34,0.62} \right)$	$\left( {0.43,0.51,0.27} \right)$
$x_{3}$	$\left( {0.52,0.36,0.48} \right)$	$\left( {0.56,0.32,0.41} \right)$	$\left( {0.44,0.56,0.78} \right)$	$\left( {0.46,0.73,0.38} \right)$	$\left( {0.50,0.32,0.29} \right)$
$x_{4}$	$\left( {0.81,0.24,0.53} \right)$	$\left( {0.65,0.52,0.37} \right)$	$\left( {0.64,0.25,0.48} \right)$	$\left( {0.28,0.52,0.47} \right)$	$\left( {0.62,0.10,0.50} \right)$
**Step 4.**: To get the normalize group T-SF decision matrix, the Eq. (21) is utilized, which is given in Table 5.

**Table 5:** Normalized group decision matrix $M_{1} = \left( {a_{ij} } \right)_{4 \times 5}$.

	$G_{1}$	$G_{2}$	$G_{2}$	$G_{4}$	$G_{5}$
$x_{1}$	$\left( {0.84,0.23,0.65} \right)$	$\left( {0.75,0.42,0.36} \right)$	$\left( {0.64,0.38,0.45} \right)$	$\left( {0.59,0.27,0.43} \right)$	$\left( {0.84,0.41,0.57} \right)$
$x_{2}$	$\left( {0.72,0.45,0.68} \right)$	$\left( {0.68,0.22,0.18} \right)$	$\left( {0.82,0.15,0.47} \right)$	$\left( {0.62,0.34,0.59} \right)$	$\left( {0.43,0.51,0.27} \right)$
$x_{3}$	$\left( {0.52,0.36,0.48} \right)$	$\left( {0.56,0.32,0.41} \right)$	$\left( {0.78,0.56,0.44} \right)$	$\left( {0.46,0.73,0.38} \right)$	$\left( {0.50,0.32,0.29} \right)$
$x_{4}$	$\left( {0.81,0.24,0.53} \right)$	$\left( {0.65,0.52,0.37} \right)$	$\left( {0.64,0.25,0.48} \right)$	$\left( {0.47,0.52,0.28} \right)$	$\left( {0.62,0.10,0.50} \right)$
**Step 5.**: The consistency measures of individual decision matrices and group decision matrix are computed by Eq. (22).
  $$CD_{1} = 0.7103$$, $$CD_{2} = 0.7322$$ $$CD_{3} = 0.7252$$.
**Step 6.**: We already set the consistency threshold $\delta = 0.72$; then, we can observe that $CD_{1} < \delta$. The consistency level of the first group of experts is low. So, we will apply the feedback mechanism to get a consensus between individuals; for that, we enter step 7.
**Step 7.**: We can see that the consistency level of the second individual is greater than that first and third, i.e. $CD_{2} > CD_{3} > CD_{1}$. So, we can use the second expert’s evaluation information as a reference to adjust the assessment information of the first expert. The revised/adjusted assessment matrix is shown in Table 6.

**Table 6:** The adjusted assessment matrix of $R^{{\left( 1 \right)\left( {l_{1} } \right)}} = \left( {a_{ij}^{\left( 1 \right)} } \right)_{4 \times 5}$.

	$G_{1}$	$G_{2}$	$G_{3}$	$G_{4}$	$G_{5}$
$x_{1}$	$\left( {0.8,0.2,0.1} \right)$	$\left( {0.7,0.2,0.5} \right)$	$\left( {0.8,0.3,0.2} \right)$	$\left( {0.7,0.6,0.2} \right)$	$\left( {0.8,0.1,0.7} \right)$
$x_{2}$	$\left( {0.7,0.2,0.6} \right)$	$\left( {0.7,0.2,0.5} \right)$	$\left( {0.5,0.1,0.4} \right)$	$\left( {0.6,0.4,0.7} \right)$	$\left( {0.8,0.1,0.5} \right)$
$x_{3}$	$\left( {0.6,0.5,0.3} \right)$	$\left( {0.5,0.4,0.6} \right)$	$\left( {0.5,0.7,0.2} \right)$	$\left( {0.5,0.1,0.4} \right)$	$\left( {0.6,0.8,0.1} \right)$
$x_{4}$	$\left( {0.8,0.1,0.7} \right)$	$\left( {0.4,0.3,0.2} \right)$	$\left( {0.4,0.3,0.6} \right)$	$\left( {0.6,0.2,0.1} \right)$	$\left( {0.7,0.1,0.4} \right)$
  Then, we return to step 2 and reapply steps 2.1, step 2.2, and step 2.3 to compute the weights of experts, which are given as follows:$w_{1} = 0.3361$, $w_{2} = 0.3322$, $w_{3} = 0.3316$.
  Now, by utilizing these weights of experts, the new group decision matrix $M_{2} = \left( {a_{ij} } \right)_{4 \times 5}$ is obtained in Table 7, and further, Table 8 gives us the normalized group decision matrix.

**Table 7:** Group decision matrix $M_{2} = \left( {a_{ij} } \right)_{4 \times 5}$.

	$G_{1}$	$G_{2}$	$G_{3}$	$G_{4}$	$G_{5}$
$x_{1}$	$\left( {0.75,0.42,0.36} \right)$	$\left( {0.64,0.38,0.45} \right)$	$\left( {0.78,0.56,0.44} \right)$	$\left( {0.52,0.43,0.78} \right)$	$\left( {0.82,0.16,0.68} \right)$
$x_{2}$	$\left( {0.62,0.10,0.50} \right)$	$\left( {0.82,0.15,0.47} \right)$	$\left( {0.52,0.36,0.48} \right)$	$\left( {0.12,0.45,0.68} \right)$	$\left( {0.28,0.40,0.62} \right)$
$x_{3}$	$\left( {0.84,0.41,0.57} \right)$	$\left( {0.81,0.24,0.53} \right)$	$\left( {0.81,0.35,0.24} \right)$	$\left( {0.42,0.45,0.23} \right)$	$\left( {0.26,0.53,0.87} \right)$
$x_{4}$	$\left( {0.47,0.52,0.28} \right)$	$\left( {0.68,0.22,0.18} \right)$	$\left( {0.22,0.36,0.65} \right)$	$\left( {0.34,0.55,0.71} \right)$	$\left( {0.86,0.30,0.42} \right)$

**Table 8:** Normalized group decision matrix $M_{2} = \left( {a_{ij} } \right)_{4 \times 5}$.

	$G_{1}$	$G_{2}$	$G_{3}$	$G_{4}$	$G_{5}$
$x_{1}$	$\left( {0.75,0.42,0.36} \right)$	$\left( {0.64,0.38,0.45} \right)$	$\left( {0.78,0.56,0.44} \right)$	$\left( {0.78,0.43,0.52} \right)$	$\left( {0.82,0.16,0.68} \right)$
$x_{2}$	$\left( {0.62,0.10,0.50} \right)$	$\left( {0.82,0.15,0.47} \right)$	$\left( {0.52,0.36,0.48} \right)$	$\left( {0.68,0.45,0.12} \right)$	$\left( {0.62,0.40,0.28} \right)$
$x_{3}$	$\left( {0.84,0.41,0.57} \right)$	$\left( {0.81,0.24,0.53} \right)$	$\left( {0.81,0.35,0.24} \right)$	$\left( {0.42,0.45,0.23} \right)$	$\left( {0.87,0.53,0.26} \right)$
$x_{4}$	$\left( {0.47,0.52,0.28} \right)$	$\left( {0.68,0.22,0.18} \right)$	$\left( {0.22,0.36,0.65} \right)$	$\left( {0.71,0.34,0.55,} \right)$	$\left( {0.86,0.30,0.42} \right)$
  Furthermore, the consistency measures of individual decision matrices and group decision matrix are computed by Eq. (22), which are given as follows:
  $$CD_{1\left( 1 \right)} = 0.7268$$, $$CD_{2\left( 2 \right)} = 0.7248$$ , $$CD_{3\left( 3 \right)} = 0.7216$$.
  We can see that $CD_{l} > \delta$, which satisfies the condition of consistency level, so $M_{2}$ is the final group decision matrix. Now we will move to the next step to get our finest alternative.
**Step 8.**: By utilizing the weights of the attributes and Eq. (9), we get the aggregated decision matrix;
  $$a_{1} = T{\text{-}}SFWPBM^{r,s} \left( {a_{11} ,a_{12} ,a_{13} ,a_{14} ,a_{15} } \right) = \left( {0.56,0.22,0.47} \right)$$
  $$a_{2} = T{\text{-}}SFWPBM^{r,s} \left( {a_{21} ,a_{22} ,a_{23} ,a_{24} ,a_{25} } \right) = \left( {0.44,0.28,0.39} \right)$$
  $$a_{3} = T{\text{-}}SFWPBM^{r,s} \left( {a_{31} ,a_{32} ,a_{33} ,a_{34} ,a_{35} } \right) = \left( {0.48,0.32,0.40} \right)$$
  $$a_{4} = T{\text{-}}SFWPBM^{r,s} \left( {a_{41} ,a_{42} ,a_{43} ,a_{44} ,a_{45} } \right) = \left( {0.53,0.27,0.43} \right)$$
**Step 9.**: By using Eq. (5), the score values are given as follows:
  $$Sc\left( {a_{1} } \right) = 0.0718$$, $$Sc\left( {a_{2} } \right) = 0.0258$$, $$Sc\left( {a_{3} } \right) = 0.0465$$, $$Sc\left( {a_{4} } \right) = 0.0693$$.
  According to the score values, the final ranking order of alternatives is $x_{1} > x_{4} > x_{3} > x_{2}$ and the obtained best alternative is $x_{1}$.

### Influence of parameters on the final result

**Figure 3:**
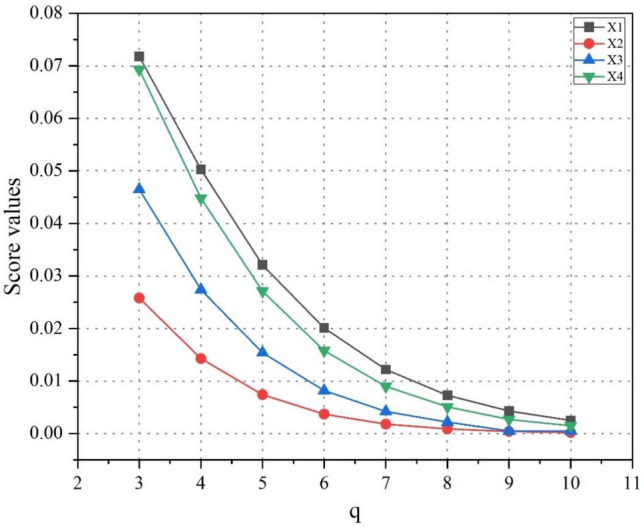
Score values of the alternatives for different values of ‘$q$’.

In some situations, when PFS and SFS are failed to deal with some information data because of their restrictions which are $MD + AD + NMD \le 1$ and $\left( {MD} \right)^{2} + \left( {AD} \right)^{2} + \left( {NMD} \right)^{2} \le 1$ respectively, then T-SFS appears up as a useful tool to deal with ambiguous and uncertain data with the condition that $\left( {MD} \right)^{q} + \left( {AD} \right)^{q} + \left( {NMD} \right)^{q} \le 1$. In this section, we analyzed the effects of different values of ‘$q$’, $r$ and $s$, on decision results to test the flexibility and sensitivity of parameters. Firstly, we observed the impact of parameter ‘$q$’ on decision results. We can see that PFSs and SFSs cannot be employed on the information data provided by experts because of the restrictions defined for these two sets. Because of this reason, we will take $3 \le q \le 10$ and the parameters ‘s’ and ‘t’ remained unchanged. The results are shown in Table 9 and graphically interpreted in Fig. 3. According to the score values obtained from using different values of parameter ‘$q$**’** and reapplying all the steps of the proposed methodology, it has been revealed that there is a change in the score values of alternatives, but the best alternative $x_{1}$ and worst alternative $x_{2}$ are the same. Also, the ranking order of the alternative by using different values of the parameter remained unchanged, which is $x_{1} > x_{4} > x_{3} > x_{2}$. One more thing is also worth noting that as the value of $q$ is increasing, the score values are decreasing. In this study, the complexity of the decision environment and conditions are represented by the value of ‘$q$’. The greater the value of ‘$q$’ means, the greater complexity is involved. To achieve appropriate decision outcomes in real-world decision problems, the experts might modify the value of ‘$q$’ in accordance with the circumstances.

**Table 9:** Influence of parameter ‘$q$’ on the ranking of alternatives.

Parameter	Score values	Ranking
$q = 3$	$SCR\left( {a_{1} } \right) = 0.0718$,$SCR\left( {a_{2} } \right) = 0.0258$, $SCR\left( {a_{3} } \right) = 0.0465$,$SCR\left( {a_{4} } \right) = 0.0693$;	$x_{1} > x_{4} > x_{3} > x_{2}$
$q = 4$	$SCR\left( {a_{1} } \right) = 0.0503$,$SCR\left( {a_{2} } \right) = 0.0143$, $SCR\left( {a_{3} } \right) = 0.0274$,$SCR\left( {a_{4} } \right) = 0.0448$;	$x_{1} > x_{4} > x_{3} > x_{2}$
$q = 5$	$SCR\left( {a_{1} } \right) = 0.0321$,$SCR\left( {a_{2} } \right) = 0.0074$, $SCR\left( {a_{3} } \right) = 0.0154$,$SCR\left( {a_{4} } \right) = 0.0271$;	$x_{1} > x_{4} > x_{3} > x_{2}$
$q = 6$	$SCR\left( {a_{1} } \right) = 0.0201$,$SCR\left( {a_{2} } \right) = 0.0037$, $SCR\left( {a_{3} } \right) = 0.0082$,$SCR\left( {a_{4} } \right) = 0.0158$;	$x_{1} > x_{4} > x_{3} > x_{2}$
$q = 7$	$SCR\left( {a_{1} } \right) = 0.0122$,$SCR\left( {a_{2} } \right) = 0.0018$, $SCR\left( {a_{3} } \right) = 0.0042$,$SCR\left( {a_{4} } \right) = 0.0090$;	$x_{1} > x_{4} > x_{3} > x_{2}$
$q = 8$	$SCR\left( {a_{1} } \right) = 0.0073$,$SCR\left( {a_{2} } \right) = 0.0009$, $SCR\left( {a_{3} } \right) = 0.0022$,$SCR\left( {a_{4} } \right) = 0.0051$;	$x_{1} > x_{4} > x_{3} > x_{2}$
$q = 9$	$SCR\left( {a_{1} } \right) = 0.0043$,$SCR\left( {a_{2} } \right) = 0.0004$, $SCR\left( {a_{3} } \right) = 0.0005$,$SCR\left( {a_{4} } \right) = 0.0027$;	$x_{1} > x_{4} > x_{3} > x_{2}$
$q = 10$	$SCR\left( {a_{1} } \right) = 0.0025$,$SCR\left( {a_{2} } \right) = 0.0002$, $SCR\left( {a_{3} } \right) = 0.0005$,$SCR\left( {a_{4} } \right) = 0.0015$;	$x_{1} > x_{4} > x_{3} > x_{2}$

**Figure 4:**
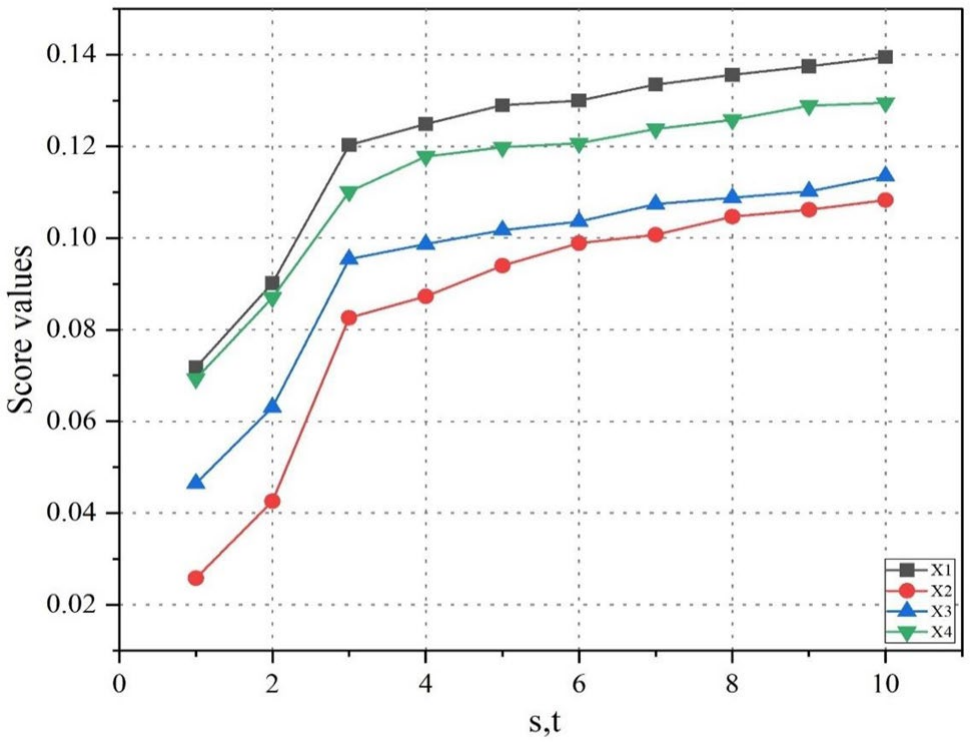
Score values of the alternatives for different values of ‘$s$’ and ‘$t$’ while ‘$q = 3$’.

Further, we take the different values of the parameters $r$ and $s$ keeping the parameter $q = 3$ unchanged. The results are shown in Table 10 and graphically interpreted in Fig. 4. It can be seen from the table that when we take the value of these parameters from 1 and 10, there a change in the score values but no change in the best and worst alternatives. Likewise, the ranking order of the alternative is also the same, which is $x_{1} > x_{4} > x_{3} > x_{2}$. It also can be observed that as the value of parameters increases, the score values of alternatives also increase.

**Table 10:** Influence of parameters ‘r’ and ‘s’ on the ranking of alternatives.

Parameter/$q = 3$	Score values	Ranking
$r = s = 1$	$SCR\left( {a_{1} } \right) = 0.0718$,$SCR\left( {a_{2} } \right) = 0.0258$, $SCR\left( {a_{3} } \right) = 0.0465$,$SCR\left( {a_{4} } \right) = 0.0693$	$x_{1} > x_{4} > x_{3} > x_{2}$
$r = s = 2$	$SCR\left( {a_{1} } \right) = 0.0902$,$SCR\left( {a_{2} } \right) = 0.0426$, $SCR\left( {a_{3} } \right) = 0.0631$,$SCR\left( {a_{4} } \right) = 0.0870$;	$x_{1} > x_{4} > x_{3} > x_{2}$
$r = s = 3$	$SCR\left( {a_{1} } \right) = 0.1203$,$SCR\left( {a_{2} } \right) = 0.0826$, $SCR\left( {a_{3} } \right) = 0.0954$,$SCR\left( {a_{4} } \right) = 0.1101$;	$x_{1} > x_{4} > x_{3} > x_{2}$
$r = s = 4$	$SCR\left( {a_{1} } \right) = 0.1249$,$SCR\left( {a_{2} } \right) = 0.0873$, $SCR\left( {a_{3} } \right) = 0.0987$,$SCR\left( {a_{4} } \right) = 0.1178$;	$x_{1} > x_{4} > x_{3} > x_{2}$
$r = s = 5$	$SCR\left( {a_{1} } \right) = 0.1290$,$SCR\left( {a_{2} } \right) = 0.0940$, $SCR\left( {a_{3} } \right) = 0.1017$,$SCR\left( {a_{4} } \right) = 0.1198$;	$x_{1} > x_{4} > x_{3} > x_{2}$
$r = s = 6$	$SCR\left( {a_{1} } \right) = 0.1300$,$SCR\left( {a_{2} } \right) = 0.0989$, $SCR\left( {a_{3} } \right) = 0.1036$,$SCR\left( {a_{4} } \right) = 0.1207$;	$x_{1} > x_{4} > x_{3} > x_{2}$
$r = s = 7$	$SCR\left( {a_{1} } \right) = 0.1335$,$SCR\left( {a_{2} } \right) = 0.1007$, $SCR\left( {a_{3} } \right) = 0.1074$, $SCR\left( {a_{4} } \right) = 0.1238$;	$x_{1} > x_{4} > x_{3} > x_{2}$
$r = s = 8$	$SCR\left( {a_{1} } \right) = 0.1356$,$SCR\left( {a_{2} } \right) = 0.1047$, $SCR\left( {a_{3} } \right) = 0.1088$,$SCR\left( {a_{4} } \right) = 0.1258$;	$x_{1} > x_{4} > x_{3} > x_{2}$
$r = s = 9$	$SCR\left( {a_{1} } \right) = 0.1375$,$SCR\left( {a_{2} } \right) = 0.1062$, $SCR\left( {a_{3} } \right) = 0.1102$,$SCR\left( {a_{4} } \right) = 0.1289$;	$x_{1} > x_{4} > x_{3} > x_{2}$
$r = s = 10$	$SCR\left( {a_{1} } \right) = 0.1395$,$SCR\left( {a_{2} } \right) = 0.1083$, $SCR\left( {a_{3} } \right) = 0.1135$,$SCR\left( {a_{4} } \right) = 0.1295$;	$x_{1} > x_{4} > x_{3} > x_{2}$

By considering the influence of parameter change, we can observe that the change in mentioned parameters $q$, $r$ and $s$ in the proposed methodology have no appreciable effect on the final decision results. The best alternative is $x_{1}$, which demonstrates the resilience of our suggested decision model and the program's ability to produce stable outcomes despite adverse environmental changes. In actual decision-making situations, the experts are advised to take the values of parameters according to the situation to get more accurate results.

### Comparative analysis

Fuzzy set^31^ can only deal with the MD of the elements and fails to deal with situations when the NMD of elements is also involved. The IFSs^34^ can tackle situations where both MD and NMD are involved. The invention of PFSs brought many applications of fuzzy set theory in decision-making issues of daily life which can deal MD, AD, and NMD. Afterward, Mahmood et al.^45^ came up with T-SFSs by improving the constraints of PFS, which cannot deal with the scenarios when the sum of MD, AD, and NMD becomes greater than one. T-SFSs allow experts to give their assessment in a broader way with minimum restrictions. Because of that reason, we used T-SFSs to get the evaluation information from experts in this study.

**Figure 5:**
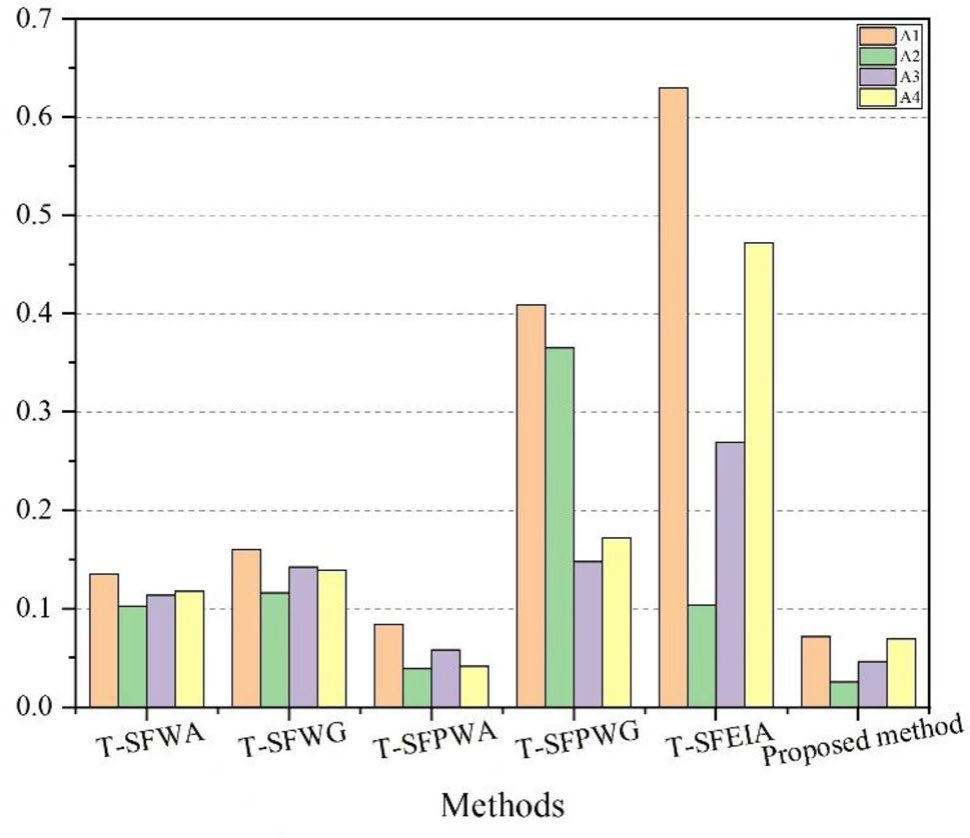
Comparison of different existing methods.

For this section, we compare our proposed approach with T-SF weighted averaging (T-SFWA) operator^60^, T-SF weighted geometric (T-SFWG) operator^60^, T-SF power weighted averaging (T-SFPWA) operator^47^, T-SF power weighted geometric (T-SFPWG) operator^47^ and T-SF Einstein interactive Averaging (T-SFEIA) operators^64^. The final results can be seen in Table 11 and graphically interpreted in Fig. 5.

**Table 11:** Ranking of alternatives with existing methods.

Methods	Score values	Ranking
T-SFWA^60^	$SCR\left( {a_{1} } \right) = 0.1356$,$SCR\left( {a_{2} } \right) = 0.1030$, $SCR\left( {a_{3} } \right) = 0.1137$,$SCR\left( {a_{4} } \right) = 0.1178$;	$x_{1} > x_{4} > x_{3} > x_{2}$
T-SFWG^60^	$SCR\left( {a_{1} } \right) = 0.1602$,$SCR\left( {a_{2} } \right) = 0.1160$, $SCR\left( {a_{3} } \right) = 0.1426$,$SCR\left( {a_{4} } \right) = 0.1389$;	$x_{1} > x_{3} > x_{4} > x_{2}$
T-SFPWA^47^	$SCR\left( {a_{1} } \right) = 0.0839$,$SCR\left( {a_{2} } \right) = 0.0390$, $SCR\left( {a_{3} } \right) = 0.0581$,$SCR\left( {a_{4} } \right) = 0.0413$;	$x_{1} > x_{3} > x_{4} > x_{2}$
T-SFPWG^47^	$SCR\left( {a_{1} } \right) = 0.4087$,$SCR\left( {a_{2} } \right) = 0.3650$, $SCR\left( {a_{3} } \right) = 0.1478$,$SCR\left( {a_{4} } \right) = 0.1725$;	$x_{1} > x_{2} > x_{4} > x_{3}$
T-SFEIA^64^	$SCR\left( {a_{1} } \right) = 0.6301$,$SCR\left( {a_{2} } \right) = 0.1039$, $SCR\left( {a_{3} } \right) = 0.2693$,$SCR\left( {a_{4} } \right) = 0.4719$;	$x_{1} > x_{4} > x_{3} > x_{2}$
Proposed method	$SCR\left( {a_{1} } \right) = 0.0718$,$SCR\left( {a_{2} } \right) = 0.0258$, $SCR\left( {a_{3} } \right) = 0.0465$,$SCR\left( {a_{4} } \right) = 0.0693$;	$x_{1} > x_{4} > x_{3} > x_{2}$

From Table 11, it has been observed that by using different existing methods, the ranking order of the alternatives is slightly different but the best alternative $x_{1}$ is the same. Furthermore, a qualitative analysis is also given in Table 12, which shows a qualitative review of the various methods discussed above. Each of these existing methods has its own advantages. However, from the above given detailed comparative analysis, it has been revealed that the technique proposed in this study is more effective, operational, and robust, which, in addition to being easy to use and calculate, may flexibly aggregate information by adjusting specific parameters to meet the preferences of experts. At the same time, the presented technique fully considers the interaction of experts and provides a feedback mechanism to get consistency degree between them to decrease the conflicts. Therefore, the designed methodology makes the evaluation outcomes of supplier selection for EMS more accurate and scientific.

**Table 12:** Qualitatively comparison of the proposed method with existing approaches.

Different methods	Depict information broadly	Aggregate information flexibly	Involve interaction of experts	Obtain the weights of experts
T-SFWA^60^	✓	✗	✗	✗
T-SFWG^60^	✓	✗	✗	✗
T-SFPWA^47^	✓	✗	✗	✗
T-SFPWG^47^	✓	✓	✗	✗
T-SFEIA^64^	✓	✓	✗	✗
Proposed method	✓	✓	✓	✓

## Conclusion

To effectively manage public emergencies and save human life, a rational and scientific evaluation of EMS suppliers is crucial. The selection of EMS suppliers is a hot research topic in MAGDM. The primary goal of this work was to create a new paradigm for efficiently addressing MAGDM problems. Firstly, some fundamental information on T-SFSs, BM operator, and PBM operator is presented. Then, T-SFPBM operator is established to integrate the individual’s information. At the same time, a method is proposed to find the experts' weights and use them to get the group decision information provided by experts. Moreover, IFM and T-SFPBM operator-based MAGDM methodology is designed to get the ranking of given alternatives. Finally, a real-life example of selecting an emergency medical supply supplier is discussed to verify the proposed approach's applicability. The stability and validity of the proposed technique are further evidenced by sensitivity analysis and comparative analysis with several existing approaches. In conclusion, it has been found that the method used in this paper is more adaptable and has a broad range to convey uncertain information. It is a suitable tool for combining vague and ambiguous data in decision-making because the provided information can be stated more precisely and definitively in it.

However, the proposed approach has been successfully applied to the selection of emergency medical supplies supplier problem, there are still some limitations that can be carried out in future studies as a continuation of this study. For example, special attention has been paid to the use of attribute weights for aggregation function in the MAGDM methods. This is a very important issue since different calculations of the attribute weights can change the ranking of possible alternatives. New methods can be proposed to get the weights of attributes. Moreover, the experts usually provide their assessment qualitatively, which we have not considered within this research.

Given the above limitations, this study presents several directions. This study can use other fuzzy decision-making methods, such as complex T-SFSs^65^, Lt-SFS^26^ and q-rung orthopair hypersoft fuzzy sets^66^, to give experts more freedom when providing their evaluation information. In this paper, the designed approach is applied to the selection of EMS suppliers during disasters. For future studies, we can apply this method to other real-world decision problems and expands its applications, such as green supplier selection^16,67,68^, medical diagnosis^69,70^, and safety risk assessment^71^.

### Informed consent

All authors agreed to this submission.

## Data Availability

The authors declare that the data supporting the findings of this study are included in this manuscript.
